# Microfluidic integrated gas sensors for smart analyte detection: a comprehensive review

**DOI:** 10.3389/fchem.2023.1267187

**Published:** 2023-09-11

**Authors:** Arian Yeganegi, Kaveh Yazdani, Nishat Tasnim, Somayeh Fardindoost, Mina Hoorfar

**Affiliations:** School of Engineering and Computer Science, University of Victoria, Victoria, BC, Canada

**Keywords:** microfluidic, gas sensors, miniaturization, selectivity, sensitivity

## Abstract

The utilization of gas sensors has the potential to enhance worker safety, mitigate environmental issues, and enable early diagnosis of chronic diseases. However, traditional sensors designed for such applications are often bulky, expensive, difficult to operate, and require large sample volumes. By employing microfluidic technology to miniaturize gas sensors, we can address these challenges and usher in a new era of gas sensors suitable for point-of-care and point-of-use applications. In this review paper, we systematically categorize microfluidic gas sensors according to their applications in safety, biomedical, and environmental contexts. Furthermore, we delve into the integration of various types of gas sensors, such as optical, chemical, and physical sensors, within microfluidic platforms, highlighting the resultant enhancements in performance within these domains.

## 1 Introduction

The air surrounding us and our exhaled breath contains many different gases which could be toxic and dangerous for human health and safety or contain markers of different diseases (Paknahad et al., 2012a; Li et al., 2014a; Yunusa et al., 2014). In recent years, the use of gas sensors for the detection and monitoring of these gases has been of great importance in environmental (Su et al., 2019a), safety monitoring (Lee et al., 2015a), and health-related (Paknahad et al., 2016a) fields.

The release of hazardous gases, such as volatile organic compounds (VOCs), ammonia, hydrogen peroxide, and petroleum gases, into the environment through different sources, including agriculture, transportation, industries, and commercial products, has deteriorated the ecosystem, changed the climate, and caused a lot of health complications, such as liver, brain, and respiratory problems (Anjum et al., 2018; Majhi et al., 2021; Narwade et al., 2021). According to the World Health Organization (WHO) report published in 2019, 91% of people worldwide reside in areas with air pollution exceeding the recommended threshold, leading to the death of approximately 4.2 million humans yearly (Buckley et al., 2020).

Gas sensors have been used widely in many safety-related applications, including public safety, household toxic gases, industries and warehouses, military and security, and food and beverage industries. Nowadays, detecting toxic and flammable gases is critical for public safety. Furthermore, as a safety measure, gas sensors are used in different industries to monitor gas leakages and are utilized in finding explosive gases (Yadav and Sinha, 2022).

The spread of numerous infectious diseases has posed a great threat to human health, and consequently, preventing the growth of these diseases has become crucial (Singh et al., 2020a). For example, gas sensors have been utilized for early diagnostics of Influenza and Ebola (Gouma et al., 2015). Monitoring and detecting more than 3,500 VOCs in the exhaled breath that can be biomarkers of different diseases is an effective use of gas sensors in healthcare applications (Dixit et al., 2021a; Kwiatkowski et al., 2018). For instance, the breath of people with diabetes, schizophrenia, and lung cancer patients frequently contains acetone, ethanol, pentane, and aldehyde (Rebordão et al., 2020a).

The conventional methods for gas sensing, like gas chromatography-mass spectroscopy, face some concerning challenges, including being bulky, expensive, time-consuming, and hard-to-operate, despite having high selectivity (Zhu et al., 2007; Barbosa et al., 2018; Rebordão et al., 2020b). This gives rise to the necessity of producing affordable, portable, selective, sensitive, and easy-to-use gas sensors, which are decent alternatives for conventional gas sensors at point-of-care and point-of-use applications. For example, by constantly monitoring human breath, these gas sensors can shed light on the cause of some respiratory diseases not directly related to environmental pollution, such as asthma, lung cancer, emphysema, and myocardial infection (Phillips et al., 1999; Kampa and Castanas, 2008). Also, they can be used in non-invasive disease diagnosis requiring low-volume biomarkers, such as dimethylsulphide and ketones, for diagnosing liver cancer and diabetes, respectively (Arasaradnam et al., 2014).

Miniaturization of gas sensors can lead to producing gas sensors with high accuracy, fast response and recovery time, low price, ease of use, and reduced analyte and reagent consumption. In this regard, microfluidic gas sensors can bring about various benefits due to their small size and the capability to integrate multiple functions into one platform, among the most important of which are being capable of *in-situ* detection, having low reagent consumption and high throughput and generating a quick response (Hong and Quake, 2003; Tani et al., 2004; Demello, 2006; Chen et al., 2019).

**FIGURE 1:**
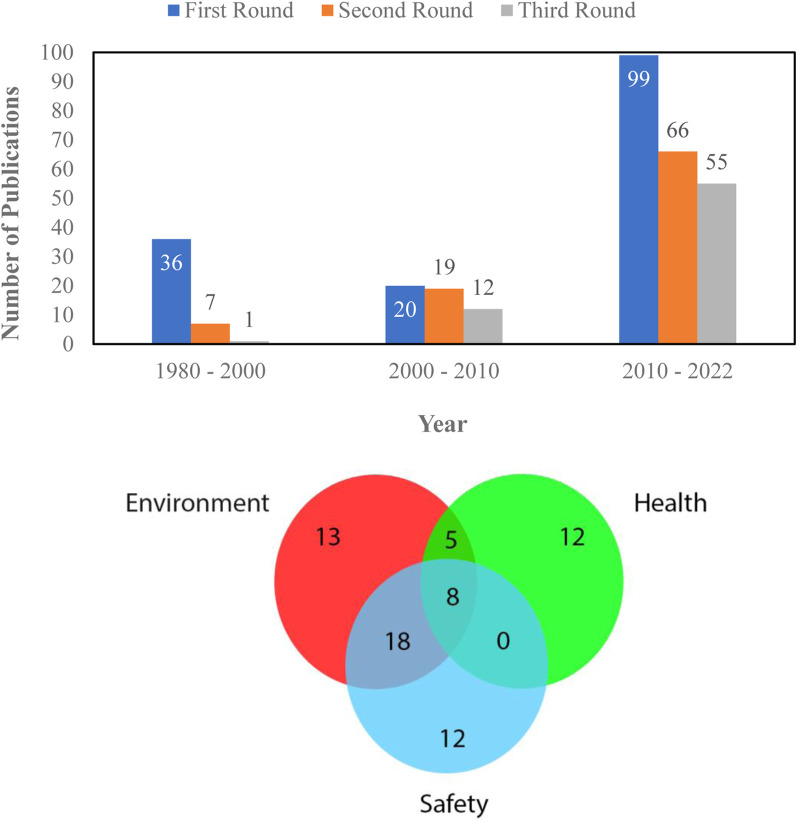
Number of papers on microfluidic gas sensor at different steps of filtration (Top) Number of papers based on application category (Bottom).

In this work, microfluidic-based gas sensors with wide applications in the environmental, safety, and health monitoring sectors have been investigated. The screening process involved selecting papers published between 1982 and the present, with a focus on the last 6 years for relevance. The papers were evaluated for their connection to gas sensor technology, particularly microfluidic-based sensors. Priority was given to reputable peer-reviewed journals and conferences in the gas sensing field. Keyword searches were conducted in databases like PubMed, Scopus, Web of Science, and Google Scholar, using terms like “gas sensors,” “microfluidic gas sensors,” and others related to monitoring applications. Studies specifically centered on microfluidic-based gas sensors were included, while those not directly relevant were excluded. Expert input was also incorporated to enhance the selection process. Papers published after 1980 were gathered and reviewed by three of the authors and categorized into the three mentioned categories. For data presentation, the authors did three rounds of data filtering. In the first round, all the papers published after 1980 and related to microfluidic gas sensing are considered. Any paper unrelated to microfluidic-based gas sensors (i.e., microfluidic-based liquid sensors), dissolved gas sensors, and all articles on microfluidic-based gas sensors published before 1980 are excluded. In the second round, each paper was reviewed by at least two of the writers and the paper was labelled as a review or technical research paper. Also, each paper was compared to our inclusion criteria, and the ones that did not meet the criteria were omitted. For the third and final round of filtration, the papers were reviewed deeply by at least two of the writers and categorized by the gas sensor application area into three different groups: environmental, personal safety, and health applications. At this step, some papers that did not meet the inclusion criteria were deleted again. Figure 1 show the number of papers gathered after each round of filtration and the number of papers based on application category, respectively.

**FIGURE 2:**
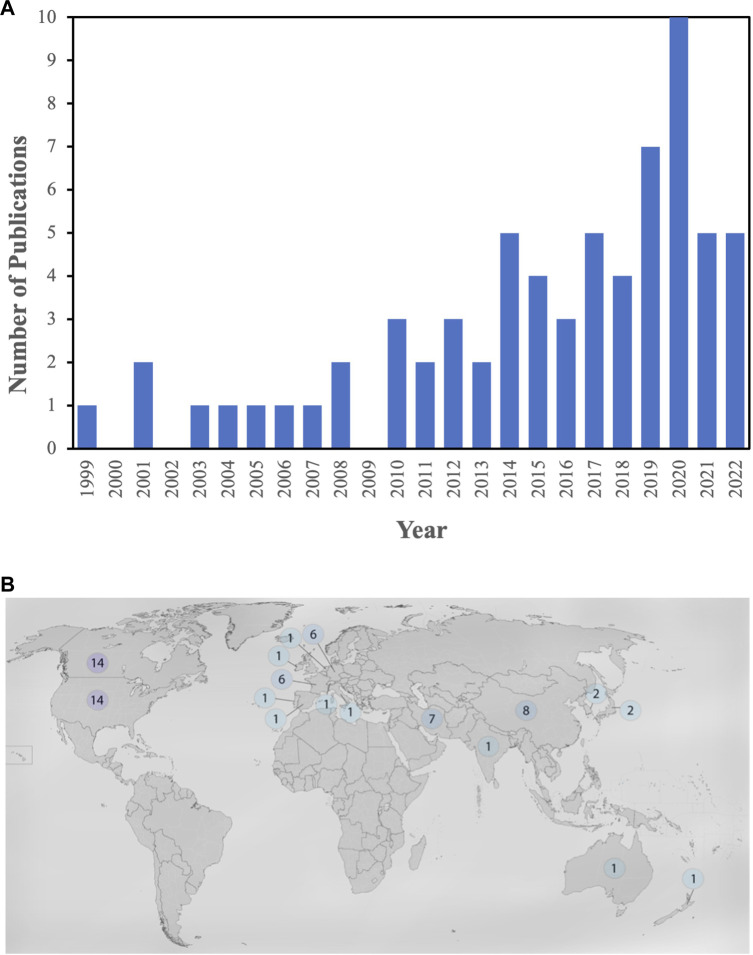
**(A)** Timeline view of the microfluidic gas sensor papers gathered for this review, **(B)** Geographical view of distribution of publications based on countries, and the correspondence authors.

Figure 2A represents the timeline view of the microfluidic gas sensor papers gathered for this review paper. As can be seen, the number of publications initially shows an increasing trend. However, after 2020, the trend changed, and the number of publications decreased. Figure 2B shows the distribution of publications based on countries and the corresponding authors. The countries with the most published papers are Canada, the United States, China, France, Germany, Iran, Japan, South Korea, Australia, India, Ireland, Italy, New Zealand, Portugal, Spain, Switzerland, and Thailand.

Essential data and information were extracted from the selected papers and tabulated, including the gas sensor detector type, target analyte, sensor fabrication, microchannel fabrication, and response results. In the beginning, the working principle of different gas sensor types and the basic associated parameters are introduced. The next section discusses the systematic search strategy, eligibility criteria, and method of categorization of the papers. The three application categories of microfluidic gas sensors are introduced, and target analytes, detector types, and fabrication techniques are reviewed for each application. To conclude, this field’s research gaps and possible future are discussed.

## 2 Microfluidic based gas sensors: methodology, working principles, and performance metrics

In this section, the methodology, working principles, and performance metrics of different types of gas sensors that can be integrated with microfluidic channels are discussed.

### 2.1 Chemiresistive gas sensors

Generally, chemiresistive sensors work based on the conductivity change upon exposure to a target gas. Under the sensing conditions (temperature and humidity), the sensing layer is exposed to airflow at its steady state. At this stage, oxygen molecules get adsorbed at the surface of the sensing material in different types depending on the working temperature. The oxygen adsorption reaction traps electrons from the conduction band of the material which is generally a semiconductor, creating an electron depletion layer and hole accumulated layer at the surface of an n-type and p-type semiconductor, respectively. As the sensing layer is exposed to a target gas, the adsorbed oxygen species react with the target gas and change the density of the electrons in the conduction band. So, the sensing layer resistance would either increase or drop depending on the type of the it (i.e., n-type or p-type) and the type of the target gas (i.e., reducing or oxidizing). By exposing back to air, the sensing layer resistance will change, until it gets back to its initial value. Generally, the response value in chemiresistive gas sensors can be obtained by investigating either the conductivity changes or changes in resistance upon exposure to a target gas. The conductivity changes can be monitored by measuring the sensing layer conductivity (G, S), resistance (R, Ω ), the current passing through (I, A), or the voltage of a series resistance (V, v), which all can be easily converted to one another. Assuming the resistance as the parameter being monitored, the response value is defined as the ratio of the sensing layer resistance at the steady state over the minimum resistance value the sensor reaches ( $\frac{R_{a}}{R_{g}}$ ) (upon exposure of an n-type sensing layer to a reducing layer or p-type sensing layer to an oxidizing gas), or *vice versa* ( $\frac{R_{g}}{R_{a}}$ ) (upon exposure of an n-type sensing layer to an oxidizing gas or p-type sensing layer to a reducing gas), where R_a_ is the resistance at the steady-state and R_g_ is the resistance upon exposure to a target gas. The response value can also be defined as the ratio of the total change in resistance over the resistance at the steady state stage ( $\frac{∆R}{R_{a}}\times 100$ ).

The principles described above are fundamental to the operation of most chemiresistive gas sensors, enabling their use in a wide range of applications for smart analyte detection. Tin Oxide (SnO_2_), Zinc Oxide (ZnO), Tungsten Oxide (WO_3_), Titanium Dioxide (TiO_2_), and Copper Oxide (CuO) are some common examples of metal oxide based chemiresistive gas sensors widely used for detecting various gases, including methane (CH_4_), carbon monoxide (CO), nitrogen dioxide (NO_2_), ammonia (NH_3_), hydrogen (H_2_), hydrogen sulfide (H_2_S) and volatile organic compounds (VOCs). These sensors operate based on the changes in the electrical conductivity of the oxide sensing layer upon exposure to the target gas. For example, in the presence of reducing gases like methane, the conductivity of the n-type oxide sensor increases, resulting in a drop in resistance. Conversely, in the presence of oxidizing gases like nitrogen dioxide, the resistance increases. These examples highlight the versatility of metal oxide semiconductor (MOS)-based gas sensors and their ability to detect various target gases through changes in their electrical conductivity (Dey, 2017; Gaun, 2020; Singh Bhati et al., 2021).

In addition to MOS, there are several other types of chemiresitive gas sensors that are widely used for detecting various gases. Nanomaterials, such as nanowires, nanotubes, and nanoclusters made from various semiconducting materials, have shown promising gas sensing properties. These nanomaterial-based sensors offer high sensitivity and selectivity for different gases. Metal-organic frameworks (MOFs) are a class of porous materials with high surface area and tunable chemical properties. MOFs can be synthesized to have selective adsorption properties for specific gases, making them useful for gas sensing applications (Sohrabi et al., 2023).

TMDs (Transition Metal Dichalcogenides) and TMTs (Transition Metal Trichalcogenides) are two types of 2D semiconductor-based gas sensors that have attracted significant research attention due to their unique properties. Their large surface-to-volume ratio and tunable bandgap make them suitable for designing high-performance and sensitive gas sensors (Kumar et al., 2020; Rafiefard et al., 2020; Shooshtari et al., 2022; Wu et al., 2023; Yang et al., 2023).

TMDs are a class of materials composed of transition metals (such as Mo, W) and chalcogen elements (such as S, Se, Te) arranged in a layered structure. They have gained prominence due to their exceptional electronic and optical properties in the 2D form. Some common TMDs include MoS_2_ (Molybdenum Disulfide), WS_2_ (Tungsten Disulfide), MoSe_2_ (Molybdenum Diselenide), and WSe_2_ (Tungsten Diselenide). TMTs are another class of 2D semiconducting materials, but they differ from TMDs in that they contain three chalcogen elements (such as S, Se, Te) for each transition metal atom. Similar to TMDs, TMTs exhibit intriguing electronic and optical properties that make them promising candidates for various applications, including sensing.

It is worth noting that the field of 2D materials and semiconductor-based sensors is rapidly evolving, and new materials and sensor configurations may have emerged.

### 2.2 Optical

Optical gas sensors work based on the adsorption properties of chemical species in different regions of the electromagnetic spectra. The region of the mid-infrared (mid-IR) spectrum spanning from 2 to 20 μm holds special significance due to its presentation of distinct vibration/rotation absorption spectra featuring narrow, distinct bands for many of the gases mentioned earlier (Bogue, 2015). Generally, the adsorption-based techniques in optical gas sensors, work based on the Beer-Lamber adsorption law which can be described as Eq. 1.

$$I=I_{0}\times \exp\left(-\alpha L\right)$$ (1)

Where $I_{0}$ and I are the intensity of the light that has passed through the cell and the intensity of the incident light, respectively. Here, α is defined as the adsorption coefficient of the gas and L is defined as the optical path length (Hodgkinson and Tatam, 2012). The adsorption-based optical methods are categorized into two main groups: direct spectrometry and reagent mediated. In direct spectrometry, the changes in a light source intensity is investigated passing through a target gas cell, whereas, in reagent mediated techniques, the sensing properties are investigated via monitoring the changes in luminousness properties of a mediated material in the presence and absence of a target gas. The response value in optical gas sensors is defined based on the type of the optical sensor, i.e., direct spectrometry and reagent mediated optical gas sensor. In direct spectrometry optical sensors, the adsorption coefficient ( α ) is defined as the response value and it can be measured via investing the intensity of incident light and emitted light. In reagent mediated optical sensors, the sensing properties are investigated by monitoring the changes in the luminescence properties of the intermediate agent, such as the refractive index, wavelength and frequency of the diffracted wave (Youssef et al., 2012), and the emission intensity (Shirbeeny and Mahmoud, 2014). Therefore, the response value could have different definitions depending on the monitored parameter. If the emission intensity is the issued parameter, as the relative change of the emission intensity, the response value can be defined as ( $\frac{I_{0}-I_{g}}{I_{0}}\times 100$ ), where $I_{0}$ and $I_{g}$ are defined as the spectra intensity in the absence and presence of the target gas, respectively (Subashini et al., 2018). Similarly, if the frequency is being monitored, the response value is defined as ( $\frac{f_{s}-f_{r}}{f_{r}}\times 100$ ), where $f_{s}$ and $f_{r}$ are defined as the frequency of the optical wavelength in the absence and presence of the target gas, respectively (Kladsomboon et al., 2018).

### 2.3 Field effect transistor (FET)

In FET-based gas sensors, the sensing layer is used as the gate or channel of the transistor. These types of gas sensors are multi-parameter sensors meaning that upon exposure to a target gas, the change in the threshold voltage ( $V_{th}$ ), transconductance ( $g_{m}$ ), field effect mobility ( FET ), or drain current ( $I_{D}$ ) can be monitored and investigated as the sensor response (Hong et al., 2021). The threshold voltage of the sensor can be defined as Eq. 2 (Jung et al., 2020).

$$V_{th}=\phi_{ms}-\frac{Q_{ox}}{C_{ox}}+2\psi_{B}-\frac{\sqrt{2qN_{D}\varepsilon_{Si}\left|2\psi_{B}\right|}}{C_{ox}}$$ (2)

Where $\phi_{ms}$ is defined as the work function difference between the gate and the channel and $Q_{ox}$ and $C_{ox}$ are defined as the oxide charge and gate-oxide capacitance, respectively. $\psi_{B}$ , $N_{D}$ , and $\varepsilon_{Si}$ are defined as surface potential at the threshold condition, doping concentration in the channel and the dielectric constant of Si , respectively. As the gas sensor is exposed to a target gas, the sensing layer reacts with the target gas and would either accept or donate electrons based on the type of the target gas (i.e., reducing or oxidizing, respectively). Then, the work function of the sensing layer changes and would shift the value of the threshold voltage which is monitored as the response (Jung et al., 2021). In FET-based gas sensors, the response can be investigated via monitoring the drain current change as the gate voltage and field effect changes upon exposure to a target gas. Therefore, the response value can be defined as ( $\frac{I_{a}-I_{g}}{I_{g}}\times 100$ ) where $I_{a}$ and $I_{g}$ are the current magnitudes upon exposure to air and the target gas, respectively (Kladsomboon et al., 2018).

### 2.4 Bubble-based gas sensing

Bubble-based gas sensing is a recent and innovative sensing mechanism to distinguish gas types both individually and in a mixture. The general concept of this method is to stream a target gas into a liquid, using a career gas, and monitor the change in the volume of the bubble upon streaming different target gases and gas mixture ratios. As the gas is streamed into the liquid, a portion of the gas is dissolved into the liquid and another portion diffuses through the liquid and forms bubbles. The diameter of these bubbles can be affected by three main parameters: gas diffusivity ( k ), gas solubility normalized by density ( Csρ ), and the ratio of the initial dissolved gas concentration over the saturation dissolved gas concentration CiCs (Bulbul et al., 2014). Therefore, based on the target gas characteristics, one of the three parameters becomes the main parameter in defining the bubble size as the gas is streamed into a liquid (Bulbul and Kim, 2015a). The response in these types of sensors is investigated via monitoring the bubble volume versus time upon injection of the target gas and any peak or sudden change in the volume of the bubble is investigated as the sensor response.

## 3 Microfluidic based gas sensors: applications, detection and fabrication

### 3.1 Environmental monitoring

The rapid growth of industry, agriculture, and transportation has culminated in releasing various harmful pollutants into the environment. Volatile Organic Compounds (VOCs) are among these hazardous pollutants. Benzene is a VOC with significant carcinogenetic characteristics, promoting the risk factor of getting diagnosed with leukemia and lymphomas (Ueno et al., 2003a). Formaldehyde, another type of carcinogenic VOC, is one of the causes of allergic diseases in people with asthma (Yu et al., 2020; on Cancer, 2004). Chlorine, which is widely used in papermaking, dyeing, printing, and producing hydrochloric acid, and phosgene, is another harmful environmental pollutant that can harm the skin and respiratory tract, even at sub-ppm concentration (Gao et al., 2008). Ammonia is one of the essential chemicals in agriculture, as it is the foundation for all nitrogen (N) fertilizers (Riva et al., 2016), and its exposure to humans can adversely affect the eyes, skin, respiratory system, and gastrointestinal system (Welch, 2006).

Because of the detrimental effects of the pollutants mentioned above, stringent regulations are applied to control their concentration. For example, the upper limit for the concentration of benzene in the United States (U. S. E. P. A. USEPA, 1999), Japan (Ministry of the Environment, 2009), and the European Union (Directive, 2000) is equal to 0.33, 1.0, and 1.6 ppb, respectively. The maximum allowable concentration of chlorine in the atmosphere that does not pose a danger is 0.3 ppm in China (Gao et al., 2008). According to the WHO, humans should not be exposed to more than 0.08 ppm concentration of formaldehyde over 30 min (Guo X.-L. et al., 2018). Pollutants can be produced either by indoor sources, such as paints, solvents, cleaning materials, and smoke, or by outdoor sources, such as agriculture, automobile exhaust, and industry. We also spend about 85 percent of our time in closed environments such as houses, schools, supermarkets, and offices (Guillam et al., 2010; Mugherli et al., 2020). With these in mind, it is of paramount importance to design a portable gas sensor with a low limit of detection. Gas chromatography and capillary electrophoresis are among the highly sensitive gas sensors that can detect harmful pollutants with a detection limit below their threshold. That being said, they are expensive, difficult to use, sedentary, and heavy, making them unsatiable options for indoor and on-site measurements. Miniaturization of gas sensors can lead to the production of gas sensors with high accuracy, fast response and recovery time, low price, ease of use, and reduced analyte and reagent consumption. Gas sensors miniaturization can be achieved by designing microfluidic gas sensors. In what follows, we delve into various types of microfluidic gas sensors utilized to detect a wide array of analytes for monitoring gases posing a threat to the environment.

A wide array of gas sensors has been developed to detect different pollutants. In this regard, some gas sensors, like MOS-based ones, can be used in microfluidic systems in various ways. For example, Becker et al. were the first researchers that suggested the concept of using MOS in a microreactor to detect environmental pollutants, including ozone (O_3_), nitrite oxides (NO and NO_2_), CO, and CH_4_, (Becker et al., 2001a). They showed that using microreactor chambers with a volume of 45 µL can enhance the selectivity and response time of the gas sensor. This type of gas sensor includes two microreactor systems. The first one is utilized to detect reducing gases, including CO and CH_4_, and the second is used to detect oxidizing gases, including O_3_, NO, and NO_2_. Each microreactor is made of a chamber containing a SnO_2_ gas sensor surrounded by opening and closing valves. Such a system can operate in constant-flow and no-flow modes. A micropump pumps the analyte into the chamber in the constant-flow mode, and the no-flow mode initiates by closing both the inlet and outlet valves. In the no-flow mode, the gas trapped in the chamber reacts with tin oxide surfaces and produces a unique signal whereby it can be specified. However, using an external pumping source in microfluidic systems has some drawbacks, including space requirement, electrical noise, and sensor response deterioration. This problem can be addressed by integrating gas pumping into the microchannel. To address this problem, Martini et al. suggested that gas flow through the microchannel can be instigated by thermal creep along the microchannel by heating the sensing element (Martini et al., 2012a). Even though designing this microfluidic gas sensor was an important step toward miniaturizing MOS-based microfluidic gas sensors, the proposed detector has drifting issues. Tackling this problem leads to the emergence of a new type of MOS-based microfluidic gas detector. These types of gas detectors classify analytes based on their diffusivity through the microchannel and their interactions with the walls through desorption and adsorption. Given that the analyte is recognized by its physical properties rather than its chemical properties, these gas sensors have great potential to avoid generating drifts.

To the best of the authors’ knowledge, Hossein-Babaei and Ghafarinia were the first researchers that utilized this concept for gas identification (Hossein-Babaei and Ghafarinia, 2010a). The designed device consists of a 50 mm × 50 μm microchannel made of borosilicate. It uses a tin oxide-based sensor to discriminate between 24 gaseous analytes with a detection limit of 500 ppm for some of the analytes, including methanol. The sensor can also estimate the compositions of binary and ternary mixtures of analytes at various concentrations. Later, Mohaghegh Montazeri et al. (2019) used COMSOL Multiphysics to develop a numerical method capable of accurately simulating diffusion, adsorption/desorption, chemical reaction, and heat and mass transfer phenomena in the MOS-based microfluidic gas sensor. This numerical method is then used in other publications (Ghazi et al., 2021a; Ghazi et al., 2022a) to improve the LOD by modifying the channel’s geometry. While diffusion-based gas detectors eliminate most of the drifts caused by chemical reactions, their responses still depend on environmental factors, including ambient temperature and humidity. In this regard, Paknahad et al. (2014) considered the sensor a multi-input-single-output system, where the analyte (methanol) concentration in air and ambient temperature and humidity are the inputs of the system, and the output is the resistance of the sensing element. They used the analysis of variances method to carry out a sensitivity analysis to find out which inputs or their possible interactions have the most pronounced effect on the output of the device. According to the results, temperature, humidity, analyte concentration, and the interaction of the last two parameters have the most impact on the device response. In addition, a regression model is used to predict the response of the device at temperature, humidity, and analyte concentration for which experimental data are not available. In another study, Paknahad et al. (2018) studied the effect of different levels of humidity, ranging from 15% to 80%, on the performance of a diffusion-based microfluidic gas detector in detecting four different analytes, including 2-pentanol, 2-pentanone, methanol, and ethanol. The results showed that the gas detector is unable to distinguish between analytes when a small change in humidity level occurs. To tackle this problem, they developed a humidity removal membrane made of different inorganic compounds, leading to a 36% improvement in the selectivity of analytes.

In addition to the miniaturization of MOS-based gas sensors, several attempts have also been made to miniaturize optical gas sensors. Ueno et al. attempted to miniaturize GC-MS by using a nanostructured material, mesoporous silicate SBA-15, as a gas chromatographer to detect VOCs, including benzene, toluene, and xylenes (BTX) (Ueno et al., 2003a). This microfluidic device consists of two Pyrex plates, namely, separation and detection plates, with a size of 3 cm × 1 cm. Mesoporous silicate is placed in a microchannel at the separation plate and functions as an adsorbent. The adsorbed components are then heated by a thin-film heater. The samples will be desorbed at different rates based on their desorption temperatures and will then be sent to the spectrometer.

Chemiluminescence (CL) is a simple, selective, and cheap detection method that does not require optical systems and light sources, making it a more straightforward alternative than GC-MS (Su et al., 2004). Gao et al. (2008) developed a microfluidic-based CL to detect chlorine gas with a detection limit of 0.2 ppm. They utilized luminol solution as a CL reagent to instigate chemical reactions required to release electromagnetic radiation. When chlorine reacts with an alkali solution, like luminol, it converts into hypochlorite ion ( $\text{Cl}O^{-}$ ) and luminal converts to an excited state of phthalate. Having returned to the ground state, luminol releases light with a wavelength of 425 nm, which will be detected by a photomultiplier tube (Marquette and Blum, 2006). The luminol also functions as an absorption reagent, eliminating the possibility of forming bubbles at liquid-gas interfaces, which can adversely affect the accuracy of the device.

Fluorescence (FL) is like CL, except a chemical reaction does not cause electromagnetic radiation. In this method, the analyte or another molecule formed by the analyte is bombarded by photons, raising electrons to the excited level. The excited electrons then undergo energy loss and generate electromagnetic radiation by which they can be identified. Becker et al. developed a microfluidic FL gas detector (20 cm × 25 cm × 15 cm) to detect formaldehyde with a detection limit of 0.08 μg m^−3^ (Becker et al., 2020). In this device, a porous interface is used to trap formaldehyde in the acetylacetone solution, where the Hantzsch reaction transforms the trapped formaldehyde into a fluorescent molecule 3,5-Diacetyl-1,4-dihydrolutidine (DDL). The formed DDL is then detected with the use of fluorescence.

The first step toward miniaturization of optical absorption spectroscopy (OAS) gas sensors was taken by Mortensen et al. (2008). They showed that the use of a photonic crystal (PC) waveguide could improve the light absorption of the analyte by instigating slow-light optical propagation in the absorption chamber. Lai et al. (2011) showed that the combined effect of slow-light propagation with the electric field intensity enhancement created by making a slot in the PC waveguide could decrease the absorption length by a factor of 1,000, making it suitable in gas sensing applications. Alternatively, Cubillas et al. (2009) suggested the use of a hollow-core photonic crystal fiber (HPCF) to decrease the absorption length, where the electric field confined in a hollow-core interacts with the analyte following through it. Ebnali-Heidari et al. (2014) improved the HPCF concept by introducing tunability to the device by utilizing optofluidic infiltration techniques. Also, Su et al. (2019b) developed an OAS microfluidic device with an on-chip detector. This device, which utilizes a waveguide integrated PbTe detector, has better response time and lower noise and can detect methane with the detection limit of 330 ppmv.

While photoionization detectors (PIDs) are non-destructive and have high sensitivity and selectivity, they are not suitable in GC and microfluidic GC because of their high response time stemming from their large ionization chamber and dead volume. Zhu et al. (2015) utilized an archimedean spiral channel GC in concert with a rapid, flow-through, and sensitive microfluidic PID to detect various VOCs, including benzene, toluene, ethylbenzene, m-Xylene, and hexane, with the detection limit of 0.2, 0.24, 0.34, 0.34, and 0.23 ppt, respectively. The flow-through design of the PID allows it to have a low dead volume ( ∼ 2 nL), and the volume of the ionization chamber is 1.3 μ L, which is over 100 smaller than conventional PIDs.

**FIGURE 3:**
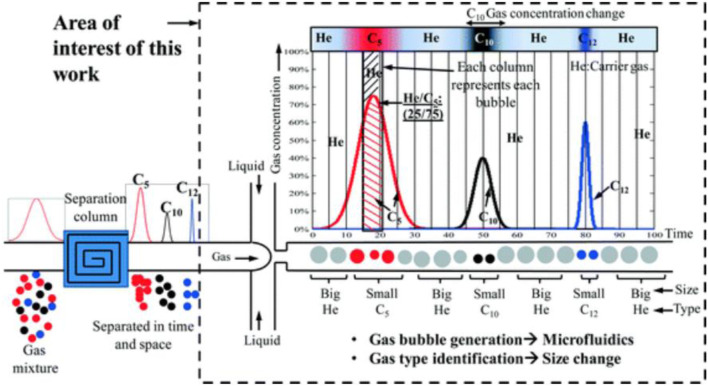
The mechanism of the bubble-based gas sensor: the gas mixture is separated into its constituents by passing through a separation column. Then, having passed through a flow-focusing microchannel, different analytes will be categorized based on their bubble diameter (Bulbul and Kim, 2015b). Reprinted with permission from RSC.

In addition to conventional MOS-based and optical gas sensors, microfluidic systems allow us to develop more innovative methods for the detection of analytes. For example, Bulbul and Kim developed a microfluidic bubble-based gas sensor to detect CO_2_, He, H_2_, CH_4,_ pentane (C_5_), and various mixtures of CO_2_—N_2_, (Bulbul and Kim, 2015b). In this method, as shown in Figure 3, helium, as an inert gas, carries a mixture through a conventional capillary column chromatographer, where the mixture is separated into its constituents. The separated gas is then transformed into a liquid channel, where bubbles will be formed because of gas-liquid interactions. Each type of gas produces bubbles with unique volumes and linear expansion coefficients, which help us to specify the type of gas.

#### 3.1.1 Fabrication methods

Several factors should be considered when it comes to the fabrication of gas sensors. For example, in MOS-based microfluidic gas sensors working based on the diffusion and adsorption of analytes, increasing the surface to volume ratio promotes the adsorption rate of the analyte to the microchannel’s walls, leading to the improvement of the sensor’s selectivity. Researchers have utilized various techniques in the fabrication process of these sensors to increase the surface-to-volume ratio. In this regard, Ghazi et al. (2021a) enhanced the selectivity of the sensor in detecting 100 ppm of seven various analytes (methanol, ethanol, propanol, pentanol, hexane, hexanal, and toluene), increasing the surface to volume ratio by decreasing the channel width and introducing nanofeatures and functional groups to the microchannel. The first part of the study, which investigates the effect of channel width on sensor selectivity, is carried out through numerical simulations, leading to an average enhancement of 93.44% and 60.1% in the selectivity of polar and non-polar VOCs, respectively. Then, the surface of the channel is modified by graphene dots, leading to an average improvement of 98.72% and 86.6% in the selective detection of polar and non-polar analytes, respectively. Finally, combining the methods mentioned above culminates in an average improvement of 101.45% and 98.82% in the selectivity for polar and non-polar analytes.

The gas sensors’ fabrication method also plays a critical role in their portability and safety. In this regard, Rezende et al. (2019) designed a microfluidic PID using micro-milling and electrical discharge machining fabrication techniques to detect toluene with the highest detection limit of 0.6 ppm. The unique fabrication of the device allows quick mount and dismount of its components. The shell of the assembly, which encapsulates all components together to minimize leakage, is made of PVC. Electrodes are fabricated by the electrical discharge machining approach and are made of copper because of its availability and high thermal conductivity. Should the electrodes be exposed to radiation higher than the operating condition of copper [4.48–4.94 eV (Haynes, 2014)], the electrons will be ejected from their surface, causing signal noise. To address this issue, the authors used two different coatings, namely, PMMA and diamond-like carbon, to filter the destructive radiations.

The new generation of smartphones provides a unique platform for implementing microfluidic technology for health monitoring (Yang et al., 2016). For example, since their digital color cameras can evaluate the color of an object, they can function as the sensor in a colorimetric gas detector. Guo X.-L. et al., 2018a developed a smartphone-based microfluidic colorimetric detector to detect formaldehyde with a detection limit of 0.01 ppm. This study chose 4-aminohydrazine-5-mercapto-1,2,4-triazole (AHMT) as the sensing reagent due to its high sensitivity, selectivity, and short response time. To prevent the fluid from coming out of the microfluidic device and allow gas samples to get in, a hydrophobic porous polytetrafluoroethylene (PTFE) membrane is utilized on the top of the reaction chamber.

**FIGURE 4:**
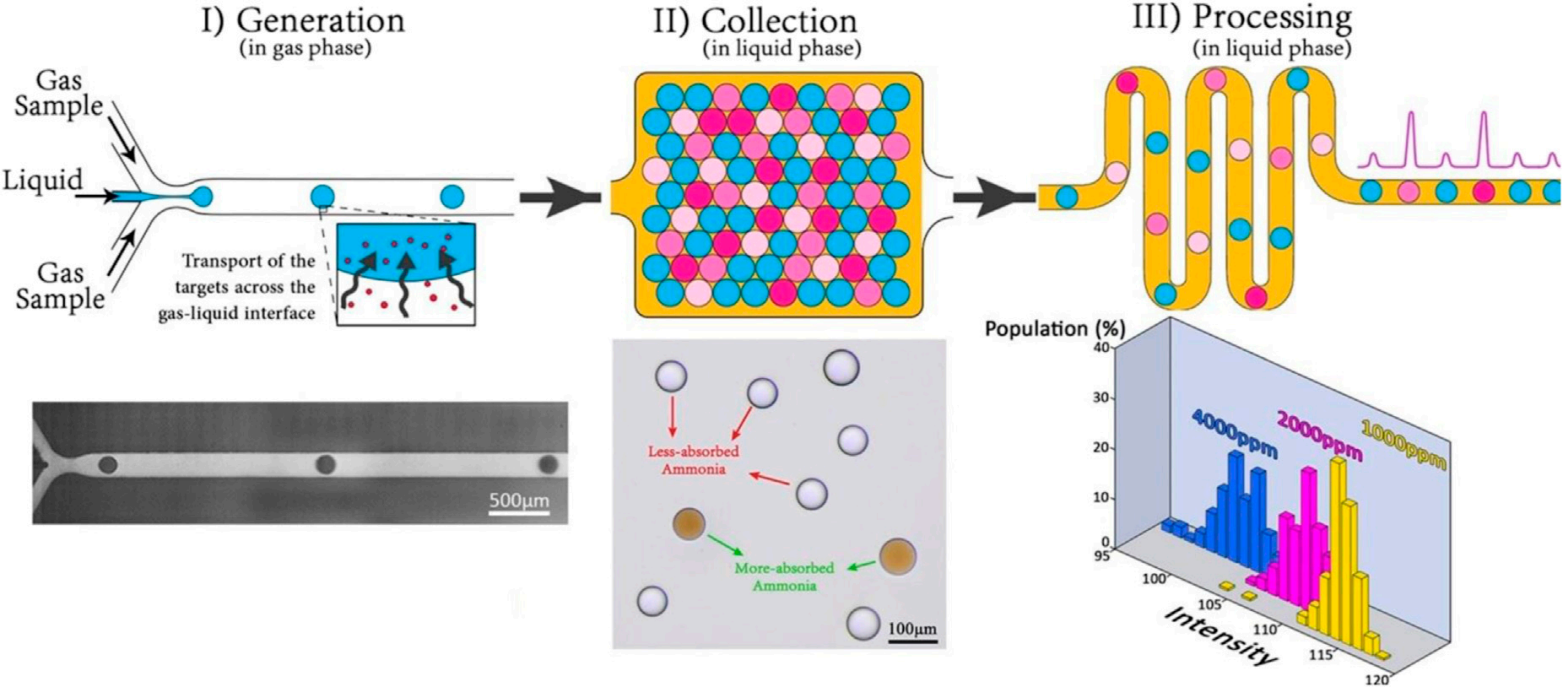
The mechanism of the colorimetric gas sensor suggested by Tirandazi and Hidrovo (Tirandazi and Hidrovo, 2018): I) The liquid phase carrying reagent enters a flow focusing microchannel, where they will be mixed with the gas analytes, II) the formed droplets containing both analytes and reagents are gathering in a liquid medium, III) The formed droplets are then carried through a series of microchannels by the liquid carrier, where further analysis is conducted on them. Reprinted with permission from Elsevier.

Innovative fabrication methods can also be used to enhance the performance of existing microfluidic devices. Tirandazi and Hidrovo fabricated a novel colorimetric device to detect airborne gaseous analytes (Tirandazi and Hidrovo, 2018). In this method, as shown in Figure 4, the liquid mixture containing the sensing reagent is injected into a continuous gas flow carrying the analyte, leading to the formation of droplets due to the interfacial tension and shear forces caused by the gaseous phase. The formed droplets are then gathered in an immiscible liquid medium, which functions as the secondary carrier. After that, the formed droplets are carried by the secondary carrier through a microchannel network, where further analysis is carried out on them. They used this method to detect vaporized ammonia (NH_3_). To that end, the liquid phase containing the Nessler reagent is injected into a mixture of dry air and ammonia. As a result of the reaction between Nessler and ammonia, the orange color spreads in the formed droplets. The colorimetric study with the detection limit of 500 ppm is then conducted using a high-speed CMOS camera (Photron SA5) coupled with a Nikon Ti-U inverted microscope as the sensor and a metallic halide white lamp as the light source. Mugherli et al. (2020) fabricated a novel FL method to detect formaldehyde. Two different molds are used to fabricate the microdevice into PDMS. The top part of the device, which is a medium through which the analyte flows, is formed using solid-film resins, and the bottom portion is made on a micro-machined brass mold. They used the sol-gel process to create the porous medium made of silica-zirconia materials. They used the anchored droplet method to generate well-calibrated micro-spheres liquid droplets with the size of 30 nL (sol phase) (Abbyad et al., 2011). Then, the porous medium is generated through the gelation of the generated droplets (gel phase). They included 4-amino-3-penten-2-one probe molecules in the sol phase, which turn into fluorescent 3,5-diacetyl-2,6-lutidine after reacting with formaldehyde.

#### 3.1.2 Analytes

Miniaturization of conventional gas detectors can extend their selectivity. For example, conventional PIDs use sealed lamps containing permanent and noble gases, such as xenon, krypton, and argon, to generate plasma that emits photons with the energy of 9.6–11.7 eV. These detectors cannot specify analytes whose ionization energy is higher than 11.7 eV, such as formaldehyde, which is one of the most important sources of environmental pollution. To tackle this problem, the researchers developed helium discharge PIDs (HDPIDs), which use helium to emit photons with the energy of 13.5–17.5 eV (Freeman and Wentworth, 1971; Gras et al., 2006; Shinada et al., 2012). However, HDPIDs’ bulky design and high helium consumption limit their application to laboratory use and make them unviable in portable GC applications. Maxwell et al. designed a microfluidic HDPID with low power and helium consumption with an in-house plasma excitation system and readout circuits (Li et al., 2021). The device was utilized to analyze permanent gases, light hydrocarbons, and formaldehyde. The limit of detection is less than 10 pg for VOCs and less than 20 pg for permanent gases, where argon with 15.76 eV ionization potential has the highest limit of detection of 19.8 pg.

The hydrophobicity of analytes plays an integral role in the selectivity of the MOS-based microfluidic detectors. In this regard, Paknahad et al. investigated the effect of channel coating hydrophobicity on the selectivity of the detector to differentiate seven different analytes with various polarities (methanol, ethanol, 1-propanol, 2-pentanol, acetone, pentane, and hexane) (Paknahad et al., 2019a). The results show that the non-polar channel coatings have better selectivity when it comes to polar analytes. This is due to the high diffusivity of polar gases, which makes diffusion the dominant factor in the diffusion-physisorption equation. With this in mind, the channel coating, which mainly affects the physisorption of the channel, has a less pronounced effect on polar analytes.

Using machine learning to differentiate between various analytes helps operators work with detectors more conveniently and faster, especially for the analytes whose responses are similar. Barriault et al. (2021) used various machine learning methods, including k-nearest-neighbors, random forests, multilayer perceptron, support vector machines, and convolutional neural networks, to discriminate between pure methane and natural gas containing 1%–3% ethane and specify the concentrations of methane and ethane in arbitrary binary mixtures of them using a MOS sensor (FIGARO, TGS 2602). According to the results, the device can distinguish between methane and natural gas with 98.75% accuracy and can specify the concentrations of methane in natural gas mixtures containing 1% and 3% ethane with an accuracy of 86.7% and 93.3%, respectively.

**FIGURE 5:**
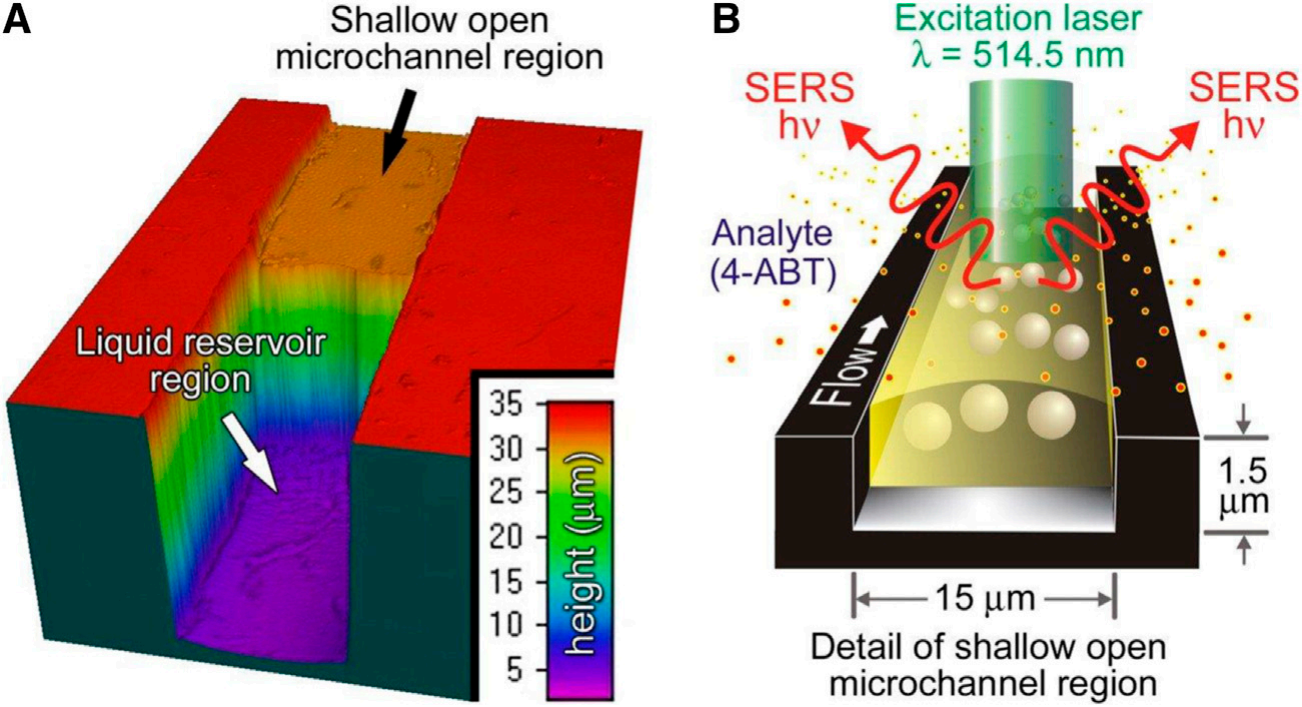
The mechanism of the microfluidic SERS gas sensor, **(A)** The carrier liquid flows from the reservoir into the open microchannel, **(B)** Au nanoparticles adsorb analytes, which leads to the formation of Au aggregates function as SERS hotspots (Piorek et al., 2007). Reprinted with permission from PNAS.

Developing a gas detector capable of detecting analytes with sub-ppm concentration is another challenge that can be addressed by using microfluidic systems. Surface-Enhanced Raman Spectroscopy (SERS) is a gas sensing method that detects analytes with femtomolar or lower concentrations (Grubisha et al., 2003). Miniaturization of these sensors helps us continuously monitor gaseous molecules released from explosions, toxic airborne compounds, and environmental contaminations (Dubnikova et al., 2005). Piorek et al. (2007) attempted to develop a microfluidic SERS gas sensor, as illustrated in Figure 5). They made a 1.5 µm × 15 µm microfluidic channel through which a free-surface flow is flowing. The small size of the microchannel provides a situation wherein the flow is confined merely by surface tension. Then, they distributed Au nanoparticles throughout the channel. The free-surface flow absorbed the analyte, namely, 4-amino benzenethiol (4-ABT). Then, the absorbed 4-ABTs diffuse through the microchannel until they adsorb onto the surface of Au nanoparticles, replacing the surface ions. The adsorbed analyte promotes the aggregation of the Au nanoparticles, forming SERS hot spots. The entrained analytes in SERS hot spots generate intense SERS spectra whereby they can be detected.

### 3.2 Personal safety monitoring

**FIGURE 6:**
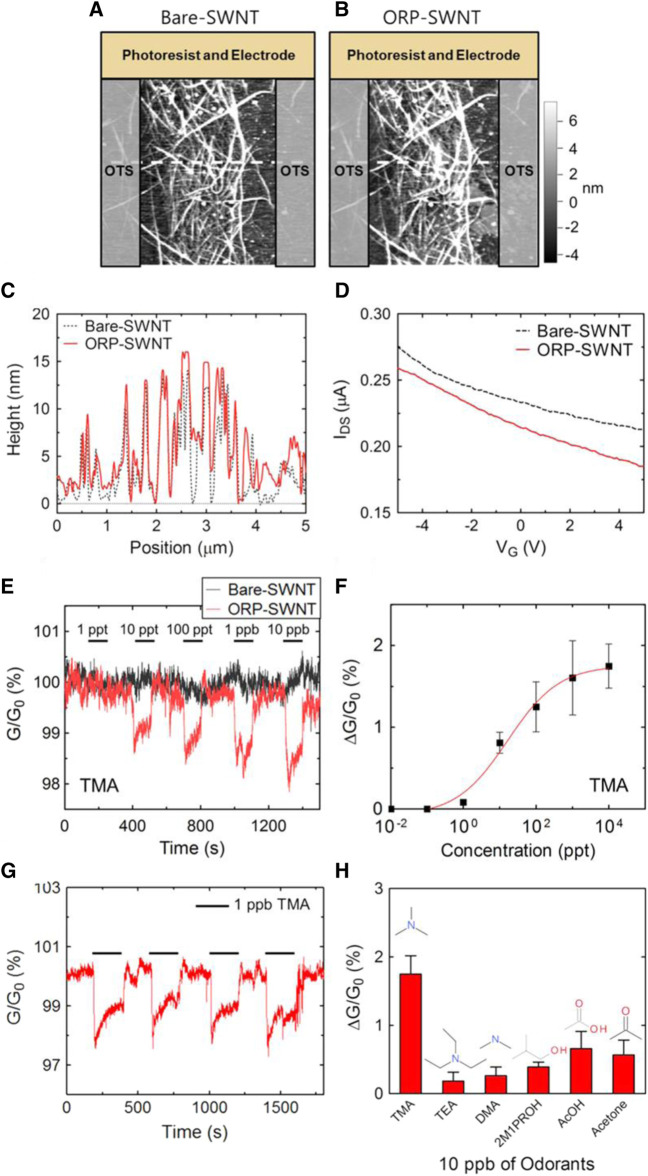
**(A–H)** The results of SWNT-FETs characterizations tests and gas sensing properties (Lee et al., 2015b). Reprinted with permission from Elsevier.

One of the leading causes of illness and mortality around the world is exposure to pollutants, toxic, and explosive compounds (Atkinson, 2000; Li et al., 2014b; Raj et al., 2015). As a result, in the last few decades many regulations have been established in order to ensure the safety of personnel, and people in different working and living areas (Bulbul et al., 2014; Bulbul and Kim, 2015a). To make sure that the established regulations and requirements are satisfied fully different sensing and monitoring devices are needed in different environments. Implementation of gas sensing and monitoring devices in a variety of living and working environments is essential to guarantee the safety of people and personnel (Youssef et al., 2012; Shirbeeny and Mahmoud, 2014). To do so, different methods and devices have been used in the past few decades such as gas chromatography and mass spectrometry (Subashini et al., 2018; Kladsomboon et al., 2018), optical-based methods (Vashist and Luong, 2018; Yu et al., 2020), acoustic-based methods (on Cancer, 2004; Gao et al., 2008), and calorimetric methods (Riva et al., 2016; Welch, 2006). Despite the advantages which these devices provide they suffer from several issues and limitations including being bulky and expensive, fabrication difficulty, risk of catalyst poisoning, risk of explosion, poor sensitivity and selectivity, need of having an expert operator, and long-term instability (Mahajan and Jagtap, 2020; Ghazi et al., 2021b). On the other hand, microfluidic gas sensors have provided compact, light, inexpensive, and user-friendly solutions for safety monitoring and as a result, they have been used excessively in this area (Ministry of the Environment, 2009; Directive, 2000). As an example, in a study, Lee et al. (2015b) fabricated a microfluidic-based gas sensor using which they were able to determine the quality of different foods and whether they are spoiled. The fabricated device can then be used to prevent illnesses caused by food poisoning. In this work, the authors developed a microfluidic-based bioelectronic nose that is able to detect gaseous trimethylamine (TMA) in a real-time and online approach (Lee et al., 2015b). Functionalized single-walled carbon nanotube-field effect transistors (SWNT-FETs) were used as the detectors of TMA which showed not only an excellent limit of detection (LOD) of 10 ppt but also a great selectivity toward TMA and other odors. The successful fabrication of SWNT-FETs and also the gas sensing properties are shown in Figures 6A–H. Microfluidic gas sensors also have potential use in the wine industry, where they could enhance the quality assessment of the wines and help rapidly identify different types of them (Paknahad et al., 2017a).

Similar to the mentioned example, there are more publications available in the literature which are proofs of successful utilization of microfluidic gas sensors for personal safety monitoring. Some of the remarkable works in the field are categorized based on the analyte type, detector type, and fabrication method which are discussed in the following sections.

#### 3.2.1 Target analytes

Different target analytes are of interest when it come to the personal safety monitoring, analytes such as sulfur-based compounds, ammonia, ozone, BTX, some ketones, and some hydrocarbons (Aliyu et al., 2015; Ghaderahmadi et al., 2021a; Song et al., 2021). Microfluidic gas sensors have shown to be promising methods for personal safety monitoring, they have been used widely for detecting and monitoring NOx (Meckes et al., 1999), VOCs such as alcohols and ketones (Janfaza et al., 2019a), hydrocarbons such as alkanes (Ghazi et al., 2021c), ammonia (Martini et al., 2012b), carbon monoxide (Meckes et al., 1999), and ozone (Becker et al., 2001b).

In a study Meckes et al. (1999) proposed a microfluidic system for gas detection. The proposed system is consisted of different channels (controlled by microvalves) and a sensing chamber (with a thin film gas sensor). The proposed setup can help improve the selectivity if the sensor and reduce the baseline drift. The authors used the setup for monitoring 100 ppm of NO and CO gases, each testing cycle is consisted of purging, calibration, and measurement steps. It has been shown that the device’s response/recovery time toward 100 ppm of NO and CO are 110/865 s and 172/844 s respectively.

**FIGURE 7:**
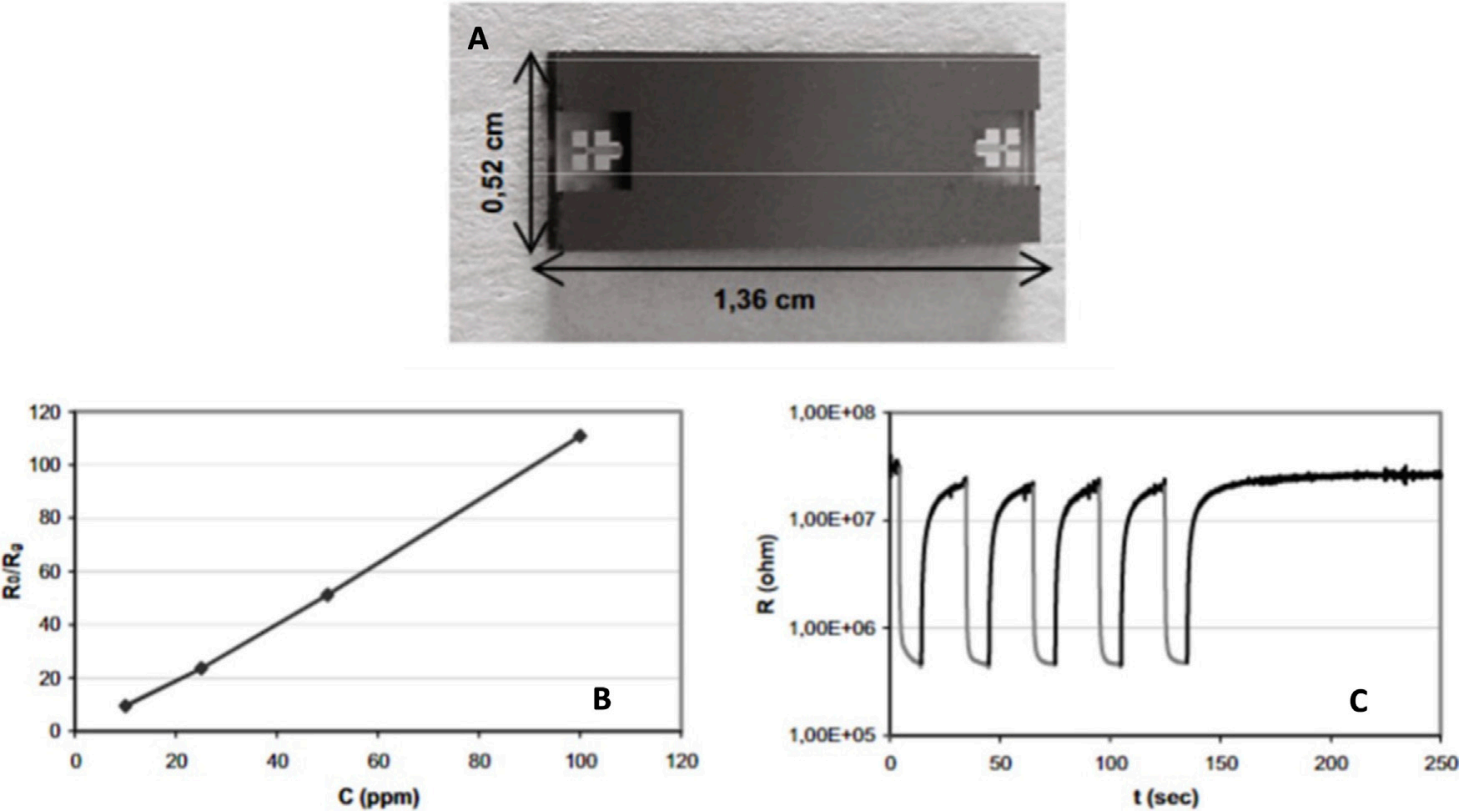
**(A)** Image of the microfluidic gas detection system, **(B)** The calibration curves, **(C)** The short term stability study of the proposed setup (Martini et al., 2010). Reprinted with permission from Elsevier.

Ammonia is among the most toxic gases and exposure to it can affect the health severely, as a result monitoring it is vital to minimize the risks. In a work (Martini et al., 2010) fabricated a gas detection microfluidic system which is capable of detecting ammonia. In this study the materials and the dimensions of the microfluidic system has been chosen based on the simulation results. Silicon and Pyrex were studied in the computational studies and based on the results Pyrex would be a better candidate than silicon. Figure 7A, shows the device with its dimensions. A MOS detector (WO_3_) is used and combined with the microchannel. The device was exposed to different concentrations of ammonia ranging from 10 to 100 ppm. Figures 7B, C show the calibration curve and short term stability of the proposed setup.

#### 3.2.2 Detector types

**FIGURE 8:**
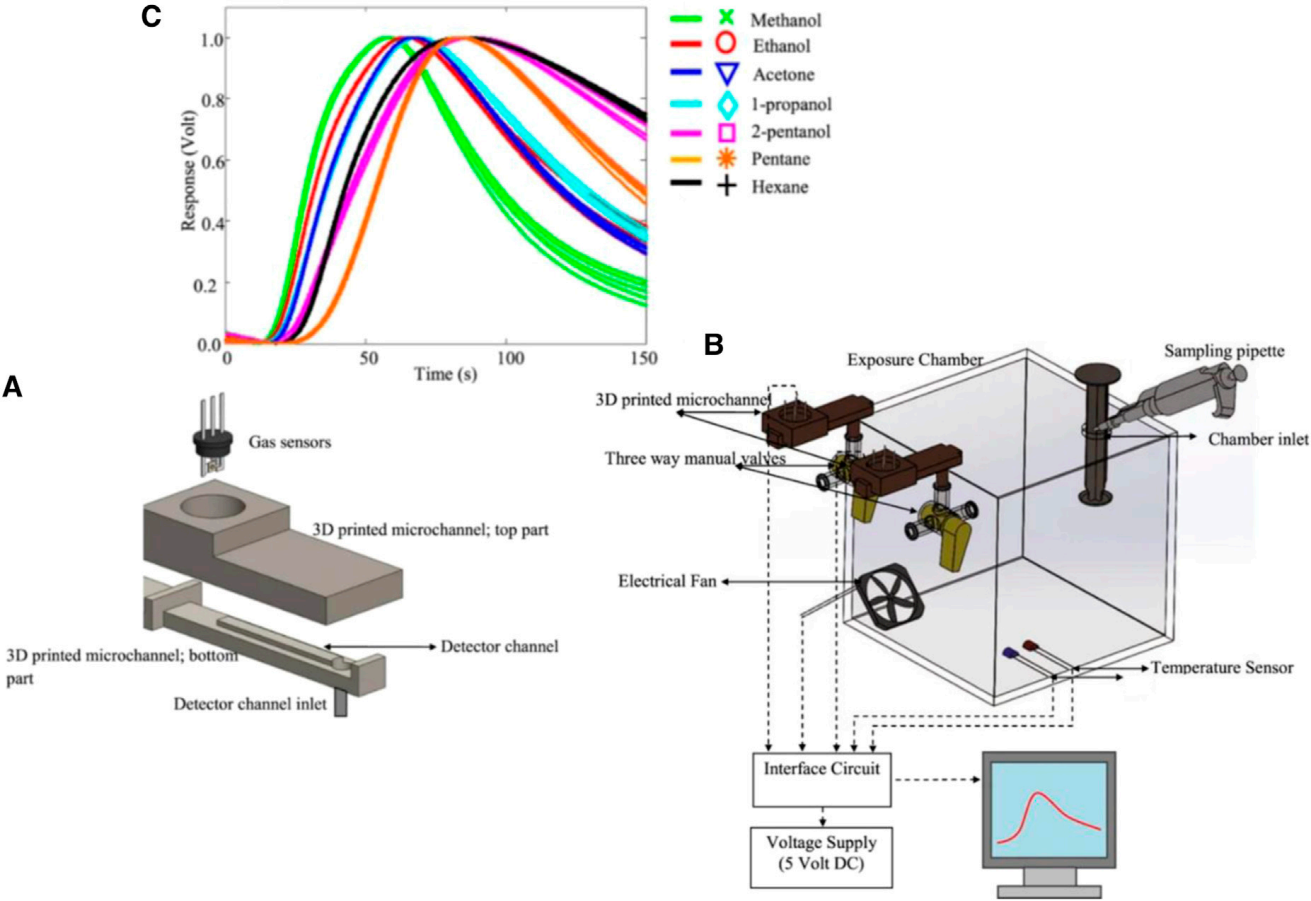
**(A)** Normalized response signals of the microfluidic gas sensor with tin oxide detector to seven different analytes with eight repetitions, **(B)** Schematic of the microfluidic gas sensor, and **(C)** The schematic of the sensing setup (Paknahad et al., 2019b). Reprinted with permission from Springer Nature.

Generally, detectors used in microfluidic gas sensors for personal safety monitoring can be categorized into three main groups including chemiresistors, optical sensors, and colorimeters (Ghazi et al., 2022b; Ueno et al., 2005a; Lee et al., 2015c). Each of these detectors provides specific advantages and disadvantages based upon which they are used for different applications with different requirements, and limitations. Among different available chemiresistors, MOS sensors have been used in microfluidic gas sensors the most. This group of sensors provides fast, and sensitive response signals with relatively low costs and ease of operation (Ghaderahmadi et al., 2021b). However, they suffer from poor selectivity, and in some conditions poor stability. Paknahad et al. (2019b) used a tin oxide semiconductor in a microfluidic gas sensor to detect 1,000 ppm of different VOCs including hexane which is known to be the cause of negative pulmonary changes. In this study the authors mounted a commercially available tin oxide detector (Figaro TGS 2602) at the end of a microfluidic channel (to improve the selectivity of the MOS sensor) and achieved and improved performance by optimizing the surface chemistry of the microchannel in terms of the polarity. It has been shown that using a non-polar coating (i.e., Cytonix on top of Parylene C) for the microchannel improves the selectivity of the sensor toward non-polar compounds such as hexane based on the “like dissolves like principle”. Normalized response signals of this sensor to different analytes and the schematic of the sensor, and sensing setup are shown in Figure 8.

**FIGURE 9:**
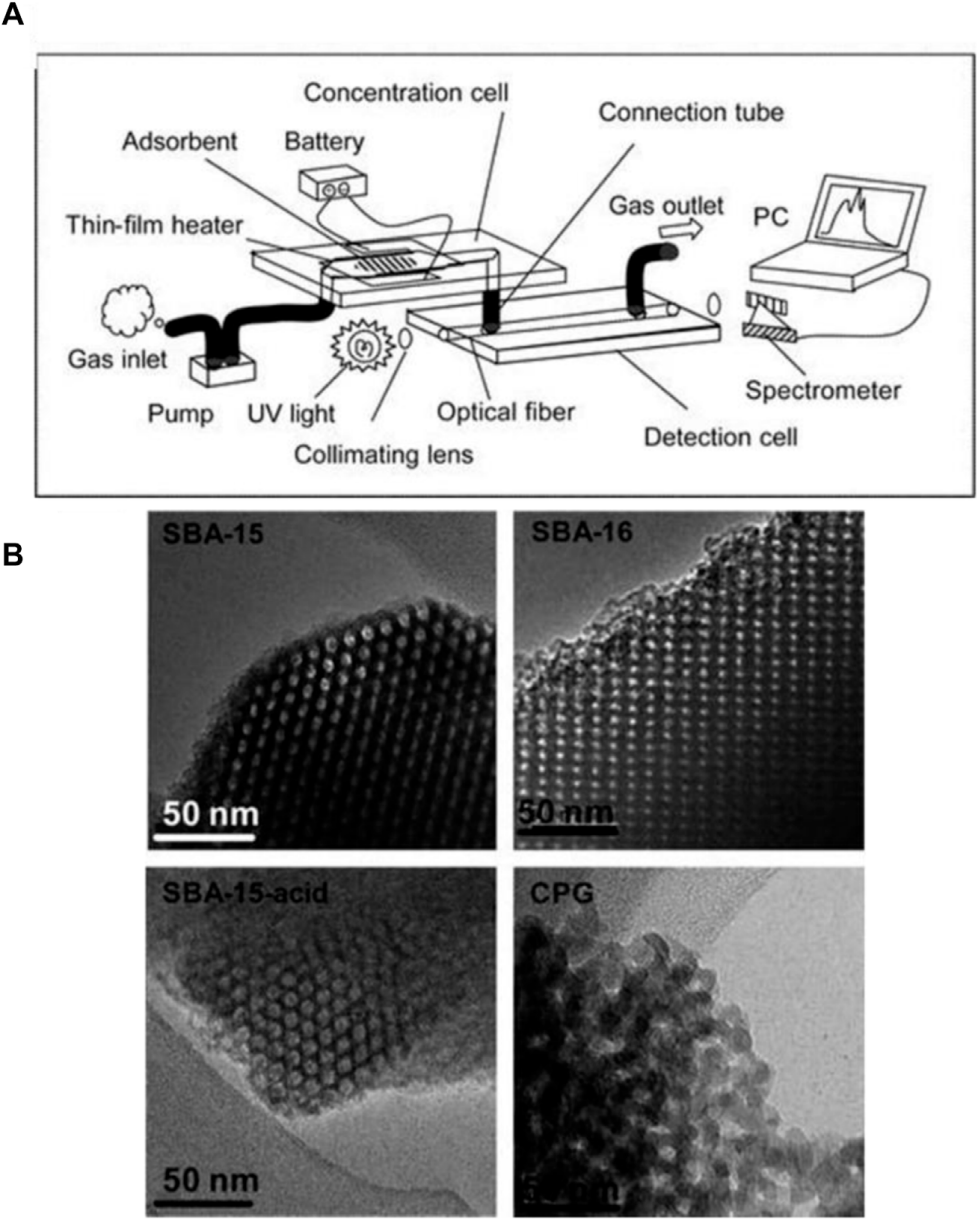
**(A)** Schematic of the gas sensing device, **(B)** TEM images of SBA-15, SBA-16, SBA-15-acid, and CPG (Chatterjee et al., 2020). Reprinted with permission from RSC.

Another group of detectors which have been widely used in microfluidic gas sensors are optical detectors. These group of detectors provide high sensitivity, long term stability, and low LOD. However, their application is limited due to a few downsides such as high cost and complexity (Liu et al., 2012). In a study Ueno et al. worked on detection of benzene, toluene, and xylene with high selectivity (Ueno et al., 2005a). The authors fabricated a concentration channel with length of 5 mm loaded with 0.5 mg of adsorbent. A microchannel with inlets and outlets is integrated into the detection unit which is aligned between a UV light source (30 W deuterium (D2) lamp, Soma Optics) and a UV spectrometer (modified Fastevert S-2400, Soma Optics). Both ends of the channel are also covered with optical fibers (Figure 9A). It is stated that by introducing mesoporous silicate (SBA16) to the microfluidic setup, a 100 ppb limit of detection of benzene with high selectivity can be achieved due to it’s uniform cubic structure (Figure 9B). In another study, Chatterjee et al. have developed an optical hydrogen gas sensor utilizing aluminum-doped zinc oxide (AZO) nanotubes inside a microchannel. After exposing their sensor to hydrogen gas at room temperature and pressure, a considerable shift in the wavelength and reduction in the reflectance intensity is reported (Chatterjee et al., 2020).

Microfluidic gas sensors that are based on nanomaterials have high sensitivity, selectivity, reliability, small size, low power consumption, and prolonged stability. Hence, these nano gas-sensitive materials have drawn significant research interest. For instance, Liu et al. have used first principles to study the potential of Mo_2_C in the detection of SF_6_ decomposition products and they demonstrate that MO_2_C can play a critical role in FET microfluidic selective gas sensing (Liu et al., 2022).

#### 3.2.3 Fabrication methods

One of the main challenges of gas sensors is detecting analytes in the order of ppb. Preconcentrators can increase the concentration of a low-volume sample by decreasing its volume. Preconentrators are made of the adsorption and desorption processes. In the former, adsorbents adsorb the analytes; in the latter, heaters heat the adsorbents to trigger the analytes to be released (Lu and Zellers, 2002). However, the conventional preconcentrators are big in size, and their desorption stage requires a substantial amount of heat and consumes a lot of power. In this regard, Dow et al. fabricated a preconcentrator microfluidic system to detect ethylene, which is the sole indicator of fruit ripening (Dow et al., 2011). The microfluidic device comprises a series of silicon trenches whose walls function as heaters, and their surfaces are loaded by carboxen 1,000 adsorbent. Silicon trenches are etched using DRIE process. Then, anodic bonding is used to compress silicon microstructures between two Pyrex glass. The device can increase the concentration of 100 ppb ethylene by 100 time, and detect it using a PID.

**FIGURE 10:**
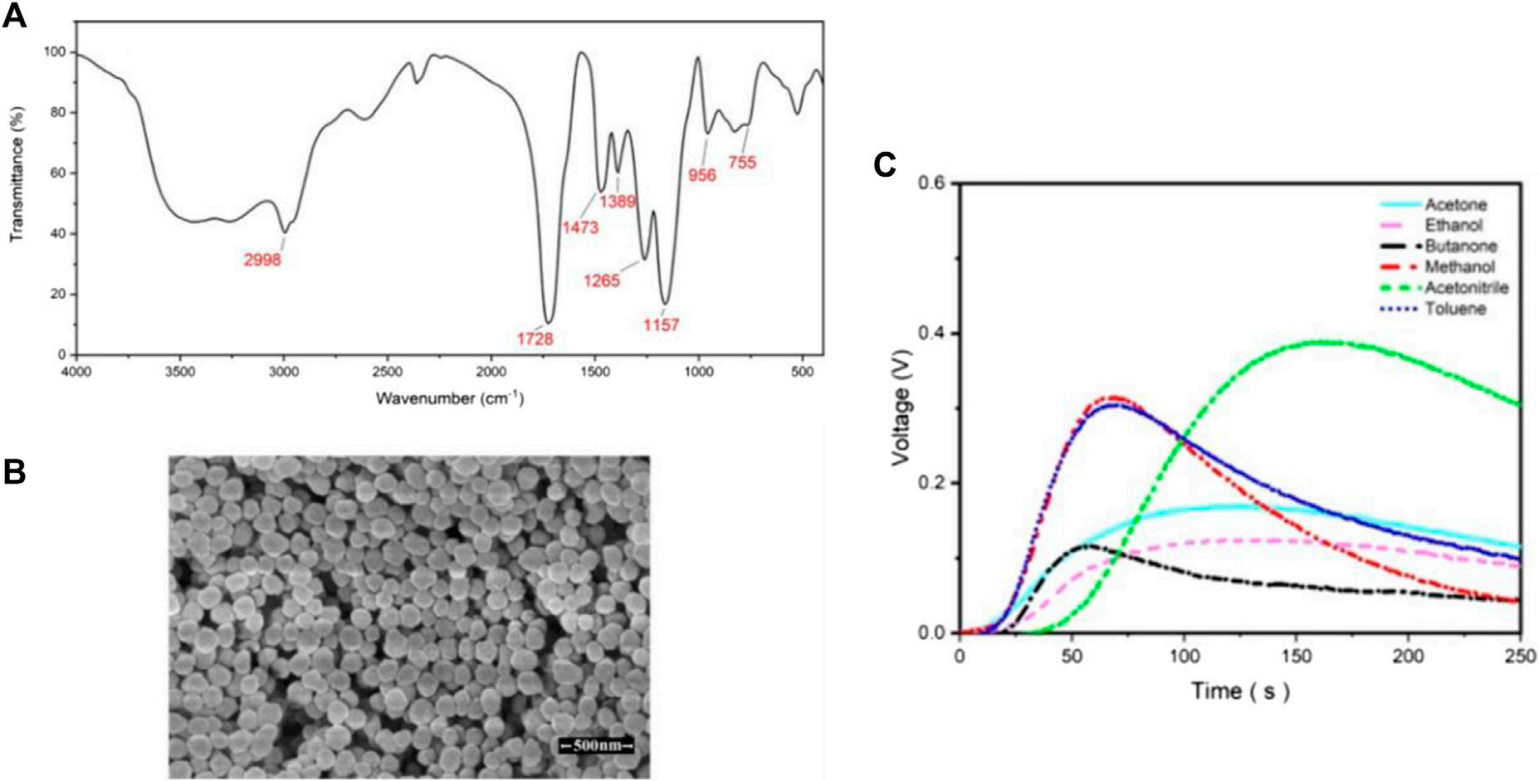
**(A)** FTIR spectra, **(B)** SEM photographs of synthesised MIP nanoparticles, **(C)** Results of the modified microfluidic gas sensor when exposed to 800 ppm of different VOCs (Janfaza et al., 2019a). Reprinted with permission from Springer Nature.

Coating the surface of microchannels is another method whereby researchers can increase the selectivity of the sensors. Molecularly imprinted polymer (MIP) is a synthetic polymer that can differentiate between different analytes due to the presence of binding sites compatible with the analytes’ size and shape. Janfaza et al. coated MIP nanoparticles with acetone recognition sites on microchannels to improve the selectivity of MOS-based microfluidic gas sensors toward different concentrations of methanol, ethanol, acetone, acetonitrile, butanone, and toluene ranging from 200 to 4,000 ppm (Janfaza et al., 2019a). A 3D printer is used to fabricate the microchannel with 500 µm height. Then, a chemical vapor deposition coating machine is used to coat the surface of the channels with Parylene C. A specified amount of MIP nanoparticles is then suspended in acetonitrile and dropcasted on the surface of the microchannel. The drying process is carried out in a clean air chamber over a day. The characterization test results are provided in Figures 10A, B and the sensor was exposed to different concentrations of different VOCs (Figure 10C)). The response values of 0.17 (vg-va), and 0.315 (vg-va) are reported when the sensor is exposed to 800 ppm of acetone and methanol respectively along with the response times of 85 and 45 s.

### 3.3 Biological related monitoring

Sensors have been utilized in many different areas, including medical and health applications, environmental monitoring, and a wide range of other industrial uses. In recent years developing devices that can sense biological parameters (biosensors) have been essential for the health industry to detect diseases in humans and cure illnesses. Biosensors have a vast area of applications (Salim and Lim, 2018; Serra, 2011). Disease monitoring, drug discovery, and the detection of contaminants, illness-causing micro-organisms, and markers that are signs of disease in bodily fluids are among the many biosensors’ applications. Among the health-related applications of biosensors, we can name cancer cell detection, cholesterol detection, glucose sensor, bacterial biosensor, pH measurement, and DNA biosensor (Antony et al., 2014a; Bhalla et al., 2016).

Biosensors are composed of three parts, a sensing material, a transducer, and an electronic system. A biosensor is categorized according to different sensing materials and transducer into various types (Singh et al., 2020b).

The goal of the healthcare industry has been the diagnosis of the disease, monitoring its propagation, and following the treatment results by implementing a non-invasive approach. Achieving this goal requires knowledge of biomarkers of different illnesses, a non-invasive technique to detect and monitor the biomarkers, and technology to distinguish the biomarkers. Diabetes, cardiovascular disease, and cancer are three of the many diseases that affect humanity significantly, and the need for an early diagnosis and a monitored treatment is crucial (Bhalla et al., 2016). Consequently, there is a significant need for a sensor to detect specific biomarkers. Gas detection and quantification, using different types of gas sensors, have been successful solutions for assessing health status (Antony et al., 2014b).

Microfluidics is becoming a widespread tool in various fields, including the gas sensor industry. Since the development of microfluidic gas sensors, they have played a crucial role in many biological and health-related applications. These devices are widely used in biomedical applications due to their excellent selectivity and sensitivity, as well as their quick response time.

#### 3.3.1 Target analytes

Different types of gas sensors have been used for health monitoring applications. Their goal is to detect certain analytes that can be useful in the detection of health problems. Methanol, ethanol, acetone, isoprene, isopropanol, propane, and undecane are of the many biomarkers that can be helpful in diagnosing diabetes, lung cancer, heart failure, and cystic fibrosis (Dixit et al., 2021b). One of the main analytes that have been studied over the years is oxygen. The ability of cells to take in oxygen is a potent indicator of sensitivity to external and internal stimuli, as well as their metabolic health and the development state of higher organisms and consequently, oxygen monitoring is of great importance (Nock et al., 2008a). Ammonia, alcohols (methanol, ethanol, and acetone), nitrogen oxide, benzene, and hydrogen have also been investigated as target analytes in the literature (Zhang et al., 2022).

Cooney and Towe (2004) have shown that in the case of using suitable sensor residence times, blood gas can be analyzed using a microdialysis-based optical sensor inside of the blood. One of the main analytes that have been studied over the years is oxygen. Bunge et al. (2019a) have studied the development of a microfluidic system to track the intake of oxygen by mammalian cells, which is a direct measure of metabolism. Their emphasis was on oxygen sensing, as well as its applicability in biolabs for large-scale cell assessments. Grist et al. (2015a) dived deep into improving sensor stability which is a must for long-term oxygen monitoring experiments. They developed a microfluidic oxygen control device with integrated ratiometric oxygen sensors to monitor chronic and cyclic Hypoxia. In another study, the patterning process was utilized to detect locally changing oxygen concentrations in both gaseous and dissolved forms by Nock et al. (2008b). Brennan et al. (2014) have successfully gathered studies on oxygen control of cells and tissues within microfluidic systems, which play a significant role in controlling oxygen tension which impacts cellular function and behavior.

Ammonia dissolved in an aqueous sample was extracted and sensed by using superhydrophobic virtual walls integrated with a microfluid chip by Raj et al. (2021a). Ammonia gas in low concentration has been measured by quantifying the electrolyte concentration in Timmer et al. (2006a) study. Paknahad et al. (2016a) developed a breath analyzer with the goal of detecting disease biomarkers. They were able to detect methanol, ethanol, and acetone in mixtures with a low concentration of 30 ppm. In another study, the effect of microchannel properties, including the channel coating and channel shape and dimensions, was studied on multiple target analytes including methanol, ethanol, and acetone (Paknahad et al., 2017b). Paknahad et al. (2012a) showed that simultaneous detection of important biomarkers such as acetone, hydrogen, benzene, and ethanol can be achieved by utilizing a system comprising a microfluidic channel with a semiconductor gas sensor. Using a novel thin film sensor with all electrochemical components combined in a multilayered, planar structure, (Cha et al., 2010) successfully created a microfluidic NO detection device which is crucial to numerous physiological processes.

#### 3.3.2 Detector types

**FIGURE 11:**
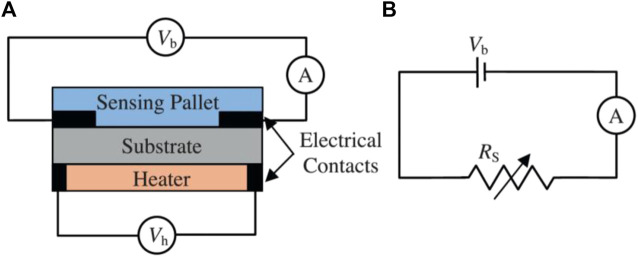
**(A)** Schematic of a basic MOS gas sensor, **(B)** Equivalent electrical circuit for the MOS sensor (Paknahad et al., 2017b). Reprinted with permission from Elsevier.

Different detector types have been employed for the detection of gases important in biological applications. The literature shows that the focus is on chemo-resistive (metal oxides) and optical gas detectors. Besides these two detection methods, electrochemical gas sensing has also been employed in a few cases. Paknahad et al. (2017b) have used tin-based metal oxide semiconductors as their sensing material for the detection of different biomarkers such as methanol, ethanol, and acetone (Figures 11A, B). This detector type is based upon the change in resistance of the sensing layer once it gets in contact with different gases (Paknahad et al., 2017b). Aghaseyedi et al. (2022) simulated gas flow in both serpentine and simple microchannels to determine the optimal microchannel configuration for enhancing gas selectivity using COMSOL Multiphysics. Through simulations they discovered that simple microchannel shows more response but less selectivity in comparison with serpentine microchannel. The optimal serpentine microchannel was fabricated and both acetone and ethanol were detected selectively and accurately using this MOS microfluidic-based gas sensor (Aghaseyedi et al., 2022).

Ammonia, an important biomarker, has been measured using two methods in the literature, optical and electrochemical methods. The optical method developed by Raj et al. (2021a) is based on gathering spectroscopic data from the sample. With aim of detecting ammonia in breath, Timmer et al. (2006a) used an electrolyte conductivity sensor besides a gas sampler and a selector. Oxygen sensing has been done using the optical method. Nock et al. (2008b) have used fluorescent dye-based optical sensing for detecting oxygen in the LOC devices application area.

#### 3.3.3 Fabrication methods

**FIGURE 12:**
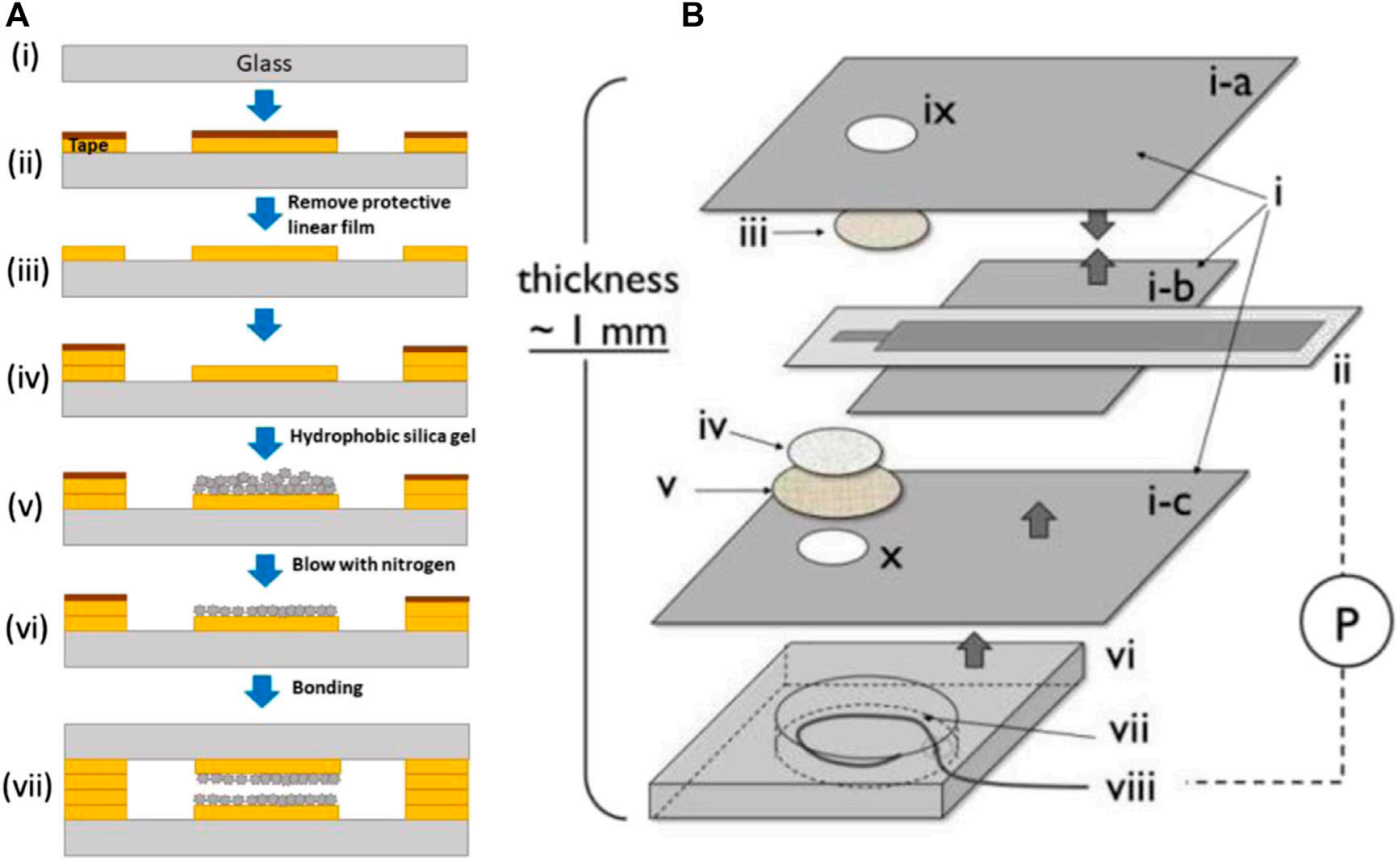
**(A)** Schematic representation of the sensor microchip fabrication (Raj et al., 2021a), **(B)** Fabrication of NO sensor (Cha et al., 2010). Reprinted with permission from ACS.

In Paknahad et al. (2017b) studies a commercial thick film metal oxide sensing pallet and a thick film thermo-resistor micro-heater are deposited on opposing surfaces of a millimetre-scale ceramic substrate to make a chemo-resistor (MOS sensor). The microchannels were either 3D printed or made using CO_2_ laser ablation and micromachining. Raj et al. (2021a) have fabricated a novel microchip using adhesive tape and adding hydrophobic silica gel. In Grate et al. (2019a) study fabrication of the oxygen sensor was done by hot embossing polystyrene against a glass-backed PDMS structure which created the pore network device in polystyrene. Nock et al. fabricated fluorescence-based oxygen sensors by using spin-coating and dry-etching methods with PDMS stamps (Raj et al., 2021a). In the process of NO sensor fabrication, 1) PE insulating tape, 2) pAu/ITO electrode, 3) TeflonAF-treated Celgard membrane, 4) Nuclepore membrane, 5) Durapore membrane filter, 6) HybriWell chamber, 7) KCl/HCl internal solution, 8) Ag/AgCl wire was used to develop a thin planar configuration (Figure 12). Also, the microchannel was fabricated using the soft lithography method (Cha et al., 2010).

## 4 Summary tables for microfluidic platforms integrated gas sesnors

Supplementary Tables S1-S3 summarize the fabricated microfluidic platforms integrated gas sesnors utilizing different detection techniques and target analytes for environment, safety, and health monitoring.

## 5 Challenges and outlook

Microfluidic integrated gas sensors have garnered significant interest in recent years due to their potential to revolutionize smart analyte detection in various applications, such as environmental monitoring, healthcare, industrial process control, and food safety. These sensors offer several advantages, including rapid analysis, miniaturization, reduced sample volumes, and the ability to detect multiple analytes simultaneously. However, they also face several challenges and issues that need to be addressed to realize their full potential. Here we cover some of the key issues, challenges, and the outlook for microfluidic integrated gas sensors.

Microfluidic gas sensors require seamless integration of different components, such as microfluidic channels, gas chambers, and sensing elements, onto a small chip. One of the primary challenges in microfluidic gas sensors is achieving high levels of miniaturization while maintaining optimal performance. Achieving high sensitivity and selectivity at low concentrations of target analytes in complex gas mixtures while discriminating against interference from other gases is one of the significant challenges. Improving the selectivity of the sensors is essential to ensure accurate and reliable analyte detection.

The other challenge is selecting appropriate materials with high chemical resistance and low gas permeability which is crucial for long-term sensor stability and accuracy. The materials used in microfluidic devices should be compatible with the target analytes to prevent chemical reactions or adsorption that could lead to false readings or sensor degradation.

For portable and battery-operated applications, minimizing power consumption is critical. Developing energy-efficient sensing mechanisms and signal processing techniques is vital to extend the sensor’s operational lifetime and reduce its environmental impact.

The widespread adoption of microfluidic gas sensors depends on their cost-effectiveness. Reducing production costs, optimizing fabrication processes, and using affordable materials are essential to make these sensors more accessible to various industries and applications. There are couple of benefits derived from utilizing microfluidic integrated gas sensors in real-world scenarios to optimize industrial processes, and improving health monitoring, resulting in reduced healthcare expenses and increased workplace safety.

Some factors to consider in relation to the economic aspects of adopting microfluidic integrated gas sensors are expenses related to the design, and manufacturing of the microfluidic integrated gas sensors associated with acquiring the necessary components such as microfluidic chips, sensing elements, electronic components, also power requirements, including power for the microfluidic systems and associated electronics, as well as expenses related to calibration to maintain sensor accuracy and reliability.

Despite the challenges, the outlook for microfluidic integrated gas sensors is promising. Continuous research and advancements in materials science, microfabrication techniques, and sensing technologies are expected to overcome many of the existing issues. The integration of microfluidic gas sensors with other smart technologies, such as wireless communication and internet of things (IoT) platforms, will enable remote and interconnected monitoring systems. This will find applications in smart cities, environmental monitoring networks, and wearable health devices. Moreover, the capability to detect multiple analytes simultaneously will further enhance the utility of microfluidic gas sensors in various industries, enabling comprehensive and multifaceted analysis.

Overall, with ongoing research and interdisciplinary collaborations, microfluidic integrated gas sensors are likely to play a significant role in the future of smart analyte detection, revolutionizing various sectors and improving the quality of life.

## 6 Conclusion

This systematic review paper focuses on microfluidic gas sensors published after 1980. It provides an overview of different types of microfluidic gas sensors and their corresponding performance metrics. The papers were categorized into three sections: environment, safety, and health, based on their respective application areas. Within each application area, the articles were analyzed with respect to detector types, target analytes, and fabrication methods.

Microfluidic gas sensors have proven valuable for environmental applications, enabling in-door and on-site measurements. They play a crucial role in ensuring the safety of individuals and workers by monitoring and detecting gases in various living and working environments. Furthermore, microfluidic gas sensors have contributed to the health sector by offering selective, sensitive, and rapid biomarker detection systems.

Conventional metal-oxide semiconductor (MOS) sensors often suffer from drift issues caused by chemical reactions. The emergence of microfluidic MOS sensors has given rise to a new generation of sensors that detect analytes based on their physical properties, potentially resolving the drifting issues. Computational fluid dynamics (CFD) simulations are utilized to optimize channel geometries by modifying dimensions, altering directions, and introducing nanofeatures. Surface coatings with substances like graphene dots and adjustment of surface polarity have also been found to impact sensor performance. Machine learning techniques have been employed in conjunction with MOS sensors to train models based on sensor data, allowing for the classification of different analytes without direct operator intervention.

In addition to MOS sensors, there has been significant interest in integrating various optical sensors, such as gas chromatography-mass spectrometry (GC-MS), chemiluminescence (CL), fluorescence (FL), optical absorption spectroscopy (OAS), photoionization detection (PID), and surface-enhanced Raman spectroscopy (SERS), into microfluidic platforms. Conventional PID sensors, like those used for formaldehyde detection, may struggle to detect analytes with high ionization energy. Microfluidic high-density PID (HDPID) sensors have been developed to address this limitation, enabling the detection of various analytes with low power consumption. Microfluidic SERS gas sensors offer continuous detection of analyte molecules released from explosions, toxic airborne compounds, and environmental contaminants at sub-ppm levels.
