# Supplementary material for: Microfluidic integrated gas sensors for smart analyte detection: a comprehensive review
Source: Front Chem. 2023 Sep 11;11:1267187. doi: 10.3389/fchem.2023.1267187 (PMC10520252; doi:10.3389/fchem.2023.1267187)
Supplement: Supplementary file 1 [file Table1.docx]

# Supplementary Tables

# Microfluidic Integrated Gas Sensors for Smart Analyte Detection: A Comprehensive Review

Arian Yeganegi^⊥^, Kaveh Yazdani ^⊥^, Nishat Tasnim, Somayeh Fardindoost, Mina Hoorfar,*

School of Engineering and Computer Science, University of Victoria, Victoria, BC, V8P 5C2,

Canada

^⊥^ These authors contributed equally

^*^ Correspondence to E-mail address: mhoorfar@uvic.ca (M. Hoorfar).

### Summary Tables for Microfluidic Platforms Integrated Gas Sensors

### Tables 1, 2, and 3 summarizes the fabricated microfluidic platforms integrated gas sensors utilizing different detection techniques and target analytes for environment, safety, and health monitoring.

Table 1. Summary of microfluidic integrated gas sensors for environmental monitoring

| Detector | Target  Analyte | Concentration | Response | Response/  Recovery times ($s$) | Limit of Detection | Sensitivity | Operating Temperature ($^{\circ}C$) | Detector fabrication | Microchannel fabrication | Reference |
| --- | --- | --- | --- | --- | --- | --- | --- | --- | --- | --- |
| MOS (SnO2) | O3, NO, NO2, CO, CH4 | O3 (40 ppb) | 2.0 ($\frac{V_{g}}{V_{a}}$) | $\sim$ 3000 s / 5000 s | - |  | 400 °C | - | Silicon micromachining Technology | [1] |
| MOS (SnO2) | 24 different analytes | 500 ppm (Methanol) | 1.8 ($\frac{V_{g}}{V_{a}}$) | $\sim50$ /95 s | - |  | 300 °C | - | - | [2] |
| MOS (WO3) | Ammonia | 25 ppm | 23 ($\frac{R_{g}}{R_{a}}$) | $\sim$ 50 / 290 s | - |  | 300 °C | Reactive radio frequency magnetron sputtering | Photolithography, lift off, etching and anodic bonding | [3] |
| MOS (SnO2) | 2-Butanol,  Isobutanol,  Tert-butanol, 1-Butanol | 250 ppm (2-Butanol) | 1.1 ($\frac{V_{g}}{V_{a}}$) | $\sim$ 75 s | - |  | 300 °C | - | Laser ablation | [4] |
| MOS (SnO2) | methanol, ethanol, 1-propanol, 2-pentanol, acetone, pentane, and hexane | 1000 ppm (2-pantanol) | 29.0 ($\frac{V_{g}}{V_{a}}$) | $\sim$ 67 s | - |  | 300 °C | - | 3D Printing | [5] |
| MOS | 2-Pentanol, Methanol, Ethanol, Acetone, 2-butanone, 2-pentanone | 250 ppm (Methanol) | 10 ($\frac{V_{g}}{V_{a}}$) | $\sim$ 50 /150 s | 250 ppm |  | 300 °C | - | 3D Printing | [6] |
| MOS | Methanol, ethanol, Propanol, Pentanol, Hexane, Hexanal, and Toluene | 100 ppm (Pentanol) | 1.3 ($\frac{R_{g}}{R_{a}}$) | $\sim$154 / 198 s | - |  | 300 °C | - | 3D Printing | [7] |
| Bubble-Based | CO2, He, H2, CH4, and C5 | 0.01 μL (C5) | 1.058 ($\frac{D_{g}}{D_{a}}$) | 25.35 s / | - |  | Room temperature | - | Oxygen plasma bonding technique | [8] |
| Photoionization detector (PID) | Benzene, toluene, ethylbenzene, m-Xylene, and hexane | 1.5 ng (Toluene) | 1.0 ($\frac{I_{g}}{I_{a}}$) | $\sim1.5$ /3 s | 0.2 ppt, 0.24 ppt, 0.34 ppt, 0.34 ppt, and 0.23 ppt |  | - | - | plasma-enhanced vapor deposition, lithography, and deep reactive-ion etching | [9] |
| PID | Toluene | 100 ppm | ∼4 nA | $\sim$ 500 ms | 40 ppb |  | 30˚C |  | micro milling and electrical discharge machining | [10] |
| PID | Several VOCs and permanent gases |  | 0.898 mV (Methane) | $\sim$ 98 ms | Less than 10 pg for VOCs and less than 20 pg for permanent gases |  |  | - | Standard lithography and deep reactive ion etching | [11] |
| Gas Chromatography-Mass Spectrometry (GC-MS)* | Benzene, Toluene, and  Xylene | Benzene (2.5 ppm)  Toluene (2.5 ppm) | 0.09 (Ig)  0.08 (Ig) | $\sim$55 min | 2.5 ppm |  | - | - | - | [12] |
| GC-MS | Benzene | 50 ppm | 0.175 (Ig) | $\sim$ 9 min | 100 ppb |  | Room temperature | - | - | [13] |
| Fabry-Pérot | CH4, C2H4, C3H6O, NH3, N2O, CO2, and He | - | 120 nm wavelength shift For C2H4 | $\sim25$/35 s | 2800 ppm (C2H4)  10,000 ppm (CO2) |  | - | - | Anodic bonding | [14] |
| Chemiluminescence | Chlorine | 478 ppm | 3250 ($I_{g}$) | Instantaneous / Instantaneous | 0.2 ppm |  | Room temperature | - | Standard photolithography and wet etching techniques | [15] |
| Fluorescence (FL) | Formaldehyde | 278 µg.m^-3^ | 4.5 ($\frac{V_{g}}{V_{a}}$) | $\sim$ 11,000 s | 0.08 µg.m-3 |  | - | - | - | [16] |
| FL | Formaldehyde | - | - | - | 10 µg/L |  | 300 °C | - | Standard Photolithography techniques | [17] |
| FL | Formaldehyde | - | - | $\sim$ 600 s | - |  | 50 °C | - | Plasma Bonding | [18] |
| Colorimetric | Formaldehyde | 0.06 ppm | 0.5 ($\frac{C_{a}}{C_{g}}$) | $\sim$18 min | 0.01 ppm |  | 18-35°C | - | Plasma Bonding | [19] |
| Optical Absorption Spectroscopy | Methane | 330 ppm | - | - | - |  | - | - | Plasma Bonding | [20] |
| Surface Enhanced Raman Spectroscopy (SERS) | 4-aminobenzenethiol (4-ABT) | 300 μM | 40000 (Ig) | 1-3 s | - |  | Room temperature | - | - | [21] |
| SERS | Benzaldehyde and 3-ethylbenzaldehyde | 1 – 100000 ppb | - | - | 1 ppb |  | Room temperature | - | Fabricated by Suzhou Wenhao Chip Tech. Co., Ltd | [22] |
| SERS | Explosives-related 2,4-dinitrotoluene (DNT), Odoriferous benzaldehyde, and Indole | 10 ppm (benzaldehyde) | - | - | 50, 10, and 1 ppm |  | Room temperature | - | Fabricated by Boston Micro Fabrication. | [23] |

Table 2. Summary of microfluidic gas sensor for the safety applications

| Detector | Target  Analyte | Concentration | Response | Response/  Recovery times ($s$) | Limit of Detection | Sensitivity | Operating Temperature ($^{\circ}C$) | Detector fabrication | Microchannel fabrication | Reference |
| --- | --- | --- | --- | --- | --- | --- | --- | --- | --- | --- |
| MOS  $SnO_{2}$ | $CO$ | 100 $ppm$ | - | 172/844 | - | -0.4 (relative) | - | rheotaxial growth and thermal oxidation | silicon micromachining | [24] |
| MOS  $SnO_{2}$ | $NO$ | 100 $ppm$ | - | 110/865 | - | -0.37 (relative) | - | rheotaxial growth and thermal oxidation | silicon micromachining | [24] |
| MOS  $SnO_{2}$ | $O_{3}$ | 40 $ppb$ | 1.39 ($\frac{V_{g}}{V_{a}}$) | 35/314 | 40 $ppb$ | - | 400 | modified rheotaxial growth and thermal oxidation | silicon micromachining | [25] |
| MOS  $SnO_{2}$ | $NO_{2}$ | 2 $ppm$ | 1.43 ($\frac{V_{g}}{V_{a}}$) | 41/1015 | 100 $ppb$ | - | 400 | modified rheotaxial growth and thermal oxidation | silicon micromachining | [25] |
| MOS  $SnO_{2}$ | $CO$ | 100 $ppm$ | 1.20  ($\frac{V_{g}}{V_{a}}$) | 45/409 | $70 ppb$ | - | 400 | modified rheotaxial growth and thermal oxidation | silicon micromachining | [25] |
| UV Spectroscopy | Benzene  $C_{6}H_{6}$ | $2.5 ppm$ | 0.94 ($\Delta i$) | 0.65/1 | - | - | - | Commercial  (30  W deuterium (D2) lamp, Soma Optics | Synthesis of mesoporous silica structures | [26] |
| UV Spectroscopy | Toluene  $C_{7}H_{8}$ | $2.5 ppm$ | 1.17 ($\Delta i$) | 1.17/1.66 | 10 $ppb$ | - | - | Commercial  (30  W deuterium (D2) lamp, Soma Optics | Synthesis of mesoporous silica structures | [26] |
| UV Spectroscopy | Xylene  $C_{8}H_{10}$ | $2.5 ppm$ | 0.87 ($\Delta i$) | 1.76/- | - | - | - | Commercial  (30  W deuterium (D2) lamp, Soma Optics | Synthesis of mesoporous silica structures  SBA-15 | [26] |
| UV Spectroscopy | Benzene  $C_{6}H_{6}$ | 50 $ppm$ | 0.01 ($\Delta i$) | - | - | -- | - | Commercial  30 W deuterium (D2) lamp, Soma Optics | Synthesis of mesoporous silica structures  SBA-16 | [27] |
| UV Spectroscopy | Toluene  $C_{7}H_{8}$ | 50 $ppm$ | 0.005 ($\Delta i$) | - | - | - | - | Commercial  30 W deuterium (D2) lamp, Soma Optics | Synthesis of mesoporous silica structures  SBA-16 | [27] |
| Optical  Ellipsometric reflection | $H_{2}$ | 4% H2 gas | 13 nm | 600/- | 0.2% H2 | - | Room temperature | Commercial ellipsometer | laser micromachining | [28] |
| Chemiresistive | Methanol | 500 $ppm$ | 1.86 $\frac{S_{g}}{S_{a}}$ | 41.36/105 | - | - | 25 | - | Generic gas sensor | [29] |
| Chemiresistive | Propanol | 900 $ppm$ | 2.10 $\frac{S_{g}}{S_{a}}$ | 68.63/- | - | - | 25 | - | Generic gas sensor | [29] |
| MOS  $WO_{3}$ | Ammonia | 50 $ppm$ | 49.4  $\frac{R_{a}}{R_{g}}$ | 5.5/8.75 |  | 1.2 | 200 | Standard microelectronic technologies | - | [30] |
| MOS  $WO_{3}$ | Ammonia | 25 $ppm$ | 27.77  $\frac{R_{a}}{R_{g}}$ | 365/1600 | - | 1 | 200 | Conventional microelectronic methods/etched in SiO2/Si | reactive radio frequency (13.56 MHz) magnetron sputtering | [31] |
| MOS  $SnO_{2}$ | Ethanol | 1000 $ppm$ | 4.18  $\frac{S_{g}}{S_{a}}$ | 51/- | - | - | 25 | made from  borosilicate glass | - (commercial) | [32] |
| ORP-coated SWNT-FETs | trimethylamine | 10 $ppb$ | 1.72  $\frac{\Delta G}{G_{0}}$ | 30/87 | 1 $ppt$ | - | Room | soft lithography and replica molding techniques with PDMS | (SWNT-FETs functionalized with olfactory receptor-derived peptides (ORPs) | [33] |
| MOS  $SnO_{2}$ | Ethanol | 1000 $ppm$ | 3.6 ($\frac{V_{g}}{V_{a}}$) | 28/68 | 10 $ppm$ | - | - | - | Commercial  SP3-AQ2, FIS Inc., Japan | [34] |
| MOS  $SnO_{2}$ | Iso-butanol | 1000 $ppm$ | 2.35 ($\frac{V_{g}}{V_{a}}$) | 29.5/80 | -- | - | - | - | Commercial  SP3-AQ2, FIS Inc., Japan | [34] |
| MOS  $SnO_{2}$ | Hydrogen Sulfide | 50 $ppm$ | 0.21 ($V_{g}-V_{a})$ | 56/- | 5 $ppm$ | - | - | 3D printer | Commercial Figaro TGS 2602 | [35] |
| MOS  $SnO_{2}$ | Methanol | 1000 $ppm$ | - | 46/>100 | - | - | - | 3D printer | Commercial Figaro TGS 2602 | [36] |
| MOS  $SnO_{2}$ | Ethanol | 1000 $ppm$ | - | 50/ >100 | - | - | - | 3D printer | Commercial Figaro TGS 2602 | [36] |
| MOS  $SnO_{2}$ | 1-Propanol | 1000 $ppm$ | - | 54/>100 | - | - | - | 3D printer | Commercial Figaro TGS 2602 | [36] |
| MOS  $SnO_{2}$ | 2- Pentanol | 1000 $ppm$ | - | 61/ >100 | - | - | - | 3D printer | Commercial Figaro TGS 2602 | [36] |
| MOS  $SnO_{2}$ | Acetone | 1000 $ppm$ | - | 52/>100 | - | - | - | 3D printer | Commercial Figaro TGS 2602 | [36] |
| MOS  $SnO_{2}$ | Pentane | 1000 $ppm$ | - | 57/>100 | - | - | - | 3D printer | Commercial Figaro TGS 2602 | [36] |
| MOS  $SnO_{2}$ | Hexane | 1000 $ppm$ | - | 66/>100 | - | - | - | 3D printer | Commercial Figaro TGS 2602 | [36] |
| MOS  $SnO_{2}$ | Acetone | 800 $ppm$ | 0.17 ${(V}_{g}-V_{a})$ | 85/>120 | - | - | 300 | MIP coated 3D printed | Commercial Figaro TGS 2600 | [37] |
| MOS  $SnO_{2}$ | Ethanol | 800 $ppm$ | 0.12  ${(V}_{g}-V_{a})$ | 90/>140 | - | - | 300 | MIP coated 3D printed | Commercial Figaro TGS 2600 | [37] |
| MOS  $SnO_{2}$ | Butanone | 800 $ppm$ | 0.11  ${(V}_{g}-V_{a})$ | 46/>190 | - | - | 300 | MIP coated 3D printed | Commercial Figaro TGS 2600 | [37] |
| MOS  $SnO_{2}$ | Methanol | 800 $ppm$ | 0.315  ${(V}_{g}-V_{a})$ | 50/>180 | - | - | 300 | MIP coated 3D printed | Commercial Figaro TGS 2600 | [37] |
| MOS  $SnO_{2}$ | Acetonitrile | 800 $ppm$ | 0.38  ${(V}_{g}-V_{a})$ | 127/>100 | - | - | 300 | MIP coated 3D printed | Commercial Figaro TGS 2600 | [37] |
| MOS  $SnO_{2}$ | Toluene | 800 $ppm$ | 0.30  ${(V}_{g}-V_{a})$ | 52/>180 | - | - | 300 | MIP coated 3D printed | Commercial Figaro TGS 2600 | [37] |
| MOS  $SnO_{2}$ | Methanol | 300 $ppm$ | 1.16  $\frac{R_{a}}{R_{g}}$ | 156/218 |  | 0.0004  $\frac{1}{ppm}$ | 300 | 3D printed  With nano and microfeatures | Commercial  Figaro TGS 2602 | [38] |
| MOS  $SnO_{2}$ | Ethanol | 300 $ppm$ | 1.23  $\frac{R_{a}}{R_{g}}$ | 172/232 |  | 0.0007  $\frac{1}{ppm}$ | 300 | 3D printed  With nano and microfeatures | Commercial  Figaro TGS 2602 | [38] |
| MOS  $SnO_{2}$ | Propanol | 300 $ppm$ | 1.28  $\frac{R_{a}}{R_{g}}$ | 209/344 |  | 0.0009  $\frac{1}{ppm}$ | 300 | 3D printed  With nano and microfeatures | Commercial  Figaro TGS 2602 | [38] |
| MOS  $SnO_{2}$ | pentnaol | 300 $ppm$ | 1.37  $\frac{R_{a}}{R_{g}}$ | 234/426 |  | 0.0012  $\frac{1}{ppm}$ | 300 | 3D printed  With nano and microfeatures | Commercial  Figaro TGS 2602 | [38] |
| MOS  $SnO_{2}$ | toluene | 300 $ppm$ | 1.22  $\frac{R_{a}}{R_{g}}$ | 295/480 |  | 0.0004  $\frac{1}{ppm}$ | 300 | 3D printed  With nano and microfeatures | Commercial  Figaro TGS 2602 | [38] |
| MOS  $SnO_{2}$ | hexanal | 300 $ppm$ | 1.47  $\frac{R_{a}}{R_{g}}$ | 184/472 | - | 0.0003  $\frac{1}{ppm}$ | 300 | 3D printed  With nano and microfeatures | Commercial  Figaro TGS 2602 | [38] |
| MOS  $SnO_{2}$ | Methanol | 100 $ppm$ | 1.26  $\frac{R_{a}}{R_{g}}$ | 89/180 | - | - | 300 | 3D printed  GQDs coated | Commercial  Figaro TGS 2602 | [39] |
| MOS  $SnO_{2}$ | Ethanol | 100 $ppm$ | 1.17  $\frac{R_{a}}{R_{g}}$ | 101/200 | - | - | 300 | 3D printed  GQDs coated | Commercial  Figaro TGS 2602 | [39] |
| MOS  $SnO_{2}$ | Propanol | 100 $ppm$ | 1.30  $\frac{R_{a}}{R_{g}}$ | 104/205 | - | - | 300 | 3D printed  GQDs coated | Commercial  Figaro TGS 2602 | [39] |
| MOS  $SnO_{2}$ | Pentanol | 100 $ppm$ | 1.54  $\frac{R_{a}}{R_{g}}$ | 121/231 | - | - | 300 | 3D printed  GQDs coated | Commercial  Figaro TGS 2602 | [39] |
| MOS  $SnO_{2}$ | Hexane | 100 $ppm$ | 1.26  $\frac{R_{a}}{R_{g}}$ | 160/>400 | - | - | 300 | 3D printed  GQDs coated | Commercial  Figaro TGS 2602 | [39] |
| MOS  $SnO_{2}$ | Hexanal | 100 $ppm$ | 2.81  $\frac{R_{a}}{R_{g}}$ | 163/332 | - | - | 300 | 3D printed  GQDs coated | Commercial  Figaro TGS 2602 | [39] |
| MOS  $SnO_{2}$ | Toluene | 100 $ppm$ | 2.70  $\frac{R_{a}}{R_{g}}$ | 160/367 | - | - | 300 | 3D printed  GQDs coated | Commercial  Figaro TGS 2602 | [39] |
| MOS  $SnO_{2}$ | NG Odorant | 10 ppm | 3.00  $\frac{R_{a}}{R_{g}}$ | 77 | 1 ppm | 0.3667 | 300 | 3D Printing | Commercial  Figaro TGS 2602 | [40] |
| MOS  $SnO_{2}$ | Hydrogen Sulphide | 10 ppm | - | 71/188 | 1 ppm | 1.026 | 300 | 3D Printing | Commercial  Figaro TGS 2602 | [40] |

Table 3. Summary of microfluidic gas sensor for the health monitoring

| Detector | Target  Analyte | Concentration | Response | Response/  Recovery times ($s$) | Limit of Detection | Sensitivity | Operating Temperature ($^{\circ}C$) | Detector fabrication | Microchannel fabrication | Reference |
| --- | --- | --- | --- | --- | --- | --- | --- | --- | --- | --- |
| spectroscopic detection | NH3 | 5 mM  ammonium hydroxide solution | - | <5 min  <300 s | - | <1 mM | - | Using Borosilicate glass and adhesive tape | - | [41] |
| Polystyrene sheet (opticalusing fluorescence image) | Oxygen | Total flow  200 mL/min | - | >0s | - | - | - | - | Polystyrene impregnated with Pt | [42] |
| Polystyrene sheet (opticalusing fluorescence image) | Oxygen | Total flow  200 mL/min |  | seconds |  |  |  |  | Polystyrene impregnated with Pt | [42] |
| Optical  silicon and glass | Oxygen | 0 - 26% (air) | - | - | ±0.8% |  | 23 - 41 °C | - | - | [43] |
| MOS  tin oxide-based | Methanol | 4000 ppm | 1.8 (vg-va) | 45s / >100s | - | - | 300 °C | Commercial (SP3-AQ2, FIS Inc., Japan) | (3D) printed | [44] |
| MOS  tin oxide-based | Ethanol | 4000 ppm | 2.05  (vg-va) | 47s/  > 100s | - | - | 300 °C | Commercial (SP3-AQ2, FIS Inc., Japan) | (3D) printed | [44] |
| MOS  tin oxide-based | Aceton | 4000 ppm | 1.8  (vg-va) | 38s/  > 100s | - | - | 300 °C | Commercial (SP3-AQ2, FIS Inc., Japan) | (3D) printed | [44] |
| Optical  Ratiometric oxygen sensor | Oxygen | 103000 ppm  (10.3% of 1 atm) | 1.93  (ratiometric R0/R) | - | - | - | - | laser-cutting patterning method | - | [45] |
| MOS | Acetone | 2000 ppm | - | 50s/  >40s | 30 ppm |  | - | Commercial | 3D-printed | [46] |
| MOS | Ethanol | 2000 ppm | - | 44s/ >45s | 30 ppm |  | - | Commercial | 3D-printed | [46] |
| MOS | Methanol | 2000 ppm | - | 39s/ > 50s | 30 ppm | - | - | Commercial | 3D-printed | [46] |
| MOS   tin oxide-based | Aceton | 3000 ppm | 1.66  (vg/va) | 54s/ >120s | - | - | 300°C | Commercial (SP3-AQ2, FIS, Japan) | CO2 laser ablation  micromachining | [47] |
| 3MOS  tin oxide-based | Hydrogen | 2500 ppm | 1.7 (vg/va) | 20s/  78s | - | - | 300°C | Commercial (SP3-AQ2, FIS, Japan) | CO2 laser ablation  micromachining | [47] |
| MOS  tin oxide-based | Ethanol | 3000 ppm | 1.58  (vg/va) | 58s/ >110s | - | - | 300°C | Commercial (SP3-AQ2, FIS, Japan) | CO2 laser ablation  micromachining | [47] |
| MOS  tin oxide-based | Benzene | 3000 ppm | 1.5  (vg/va) | 200s/ - | - | - | 300°C | Commercial (SP3-AQ2, FIS, Japan) | CO2 laser ablation  micromachining | [47] |
| MOS   tin oxide-based | Aceton | 3000 ppm | 1.66  (vg/va) | 54s/ >120s | - | - | 300°C | Commercial (SP3-AQ2, FIS, Japan) | CO2 laser ablation  micromachining | [47] |
| MOS  ZnO | $Ethanol$ | 2.6 ppm | 1.005 | 58/- | 2.6 ppm | - | 27 | sol–gel method | 3D printing | [48] |
| MOS  ZnO | $Acetone$ | 2.6 ppm | 1.004 | 50/- | 2.6 ppm | - | 27 | sol–gel method | 3D printing | [48] |
| Optical  fluorescent dye-based | Oxygen | - | 2.135  (I0/I) | 1s  nearly instantaneous/ 3s | - | - | 37 °C | Spin-coating and dry-etching with PDMS stamps | Replica modeling | [49] |
| Electrochemical  electrolyte conductivity sensor | Ammonia | 224 ppb | 3.5  (G/G0) | 96s | 1.1 ppb | - | 20 °C | photolithography | photolithography and powder blasting | [50] |

# References

[1] T. Becker, S. Mühlberger, C. Bosch-v.Braunmühl, G. Müller, A. Meckes, and W. Benecke, “Microreactors and microfluidic systems: an innovative approach to gas sensing using tin oxide-based gas sensors,” *Sens Actuators B Chem*, vol. 77, no. 1, pp. 48–54, 2001, doi: https://doi.org/10.1016/S0925-4005(01)00671-2.

[2] F. Hossein-Babaei and V. Ghafarinia, “Gas analysis by monitoring molecular diffusion in a microfluidic channel,” *Anal Chem*, vol. 82, no. 19, pp. 8349–8355, 2010.

[3] V. Martini, S. Bernardini, M. Bendahan, K. Aguir, P. Perrier, and I. Graur, “Microfluidic gas sensor with integrated pumping system,” *Sens Actuators B Chem*, vol. 170, pp. 45–50, 2012, doi: https://doi.org/10.1016/j.snb.2011.01.011.

[4] F. Hossein-Babaei, M. Paknahad, and V. Ghafarinia, “A miniature gas analyzer made by integrating a chemoresistor with a microchannel,” *Lab Chip*, vol. 12, no. 10, pp. 1874–1880, 2012.

[5] M. Paknahad, C. Mcintosh, and M. Hoorfar, “Selective detection of volatile organic compounds in microfluidic gas detectors based on ‘like dissolves like,’” *Sci Rep*, vol. 9, no. 1, pp. 1–11, 2019.

[6] M. Paknahad, J. S. Bachhal, A. Ahmadi, and M. Hoorfar, “Characterization of channel coating and dimensions of microfluidic-based gas detectors,” *Sens Actuators B Chem*, vol. 241, pp. 55–64, 2017.

[7] M. Ghazi *et al.*, “Enhanced selectivity of microfluidic gas sensors by modifying microchannel geometry and surface chemistry with graphene quantum dots,” *Sens Actuators B Chem*, vol. 342, p. 130050, 2021.

[8] A. Bulbul and H. Kim, “A bubble-based microfluidic gas sensor for gas chromatographs,” *Lab Chip*, vol. 15, no. 1, pp. 94–104, 2015.

[9] H. Zhu *et al.*, “Flow-through microfluidic photoionization detectors for rapid and highly sensitive vapor detection,” *Lab Chip*, vol. 15, no. 14, pp. 3021–3029, 2015, doi: 10.1039/C5LC00328H.

[10] G. C. Rezende, S. Le Calvé, J. J. Brandner, and D. Newport, “Characterization of a modular microfluidic photoionization detector,” *Sens Actuators B Chem*, vol. 324, p. 128667, 2020, doi: https://doi.org/10.1016/j.snb.2020.128667.

[11] M. W.-H. Li, A. Ghosh, R. Sharma, H. Zhu, and X. Fan, “Integrated microfluidic helium discharge photoionization detectors,” *Sens Actuators B Chem*, vol. 332, p. 129504, 2021, doi: https://doi.org/10.1016/j.snb.2021.129504.

[12] Y. Ueno, T. Horiuchi, O. Niwa, H. Zhou, T. Yamada, and I. Honma, “Improvement of on-site microfluidic benzene, toluene, xylene (BTX) gas sensor loaded with nanostructured mesoporous silicate,” *Sensors and materials*, vol. 15, no. 8, pp. 393–402, 2003.

[13] Y. Ueno, A. Tate, O. Niwa, H.-S. Zhou, T. Yamada, and I. Honma, “High benzene selectivity of mesoporous silicate for BTX gas sensing microfluidic devices,” *Anal Bioanal Chem*, vol. 382, no. 3, pp. 804–809, 2005, doi: 10.1007/s00216-004-2974-6.

[14] J. Tao *et al.*, “A Microfluidic-Based Fabry-Pérot Gas Sensor,” *Micromachines (Basel)*, vol. 7, no. 3, p. 36, 2016.

[15] Z.-X. Gao, H.-F. Li, J. Liu, and J.-M. Lin, “A simple microfluidic chlorine gas sensor based on gas–liquid chemiluminescence of luminol-chlorine system,” *Anal Chim Acta*, vol. 622, no. 1, pp. 143–149, 2008, doi: https://doi.org/10.1016/j.aca.2008.05.067.

[16] A. Becker, C. Andrikopoulou, P. Bernhardt, C. Trocquet, and S. Le Calvé, “On-Line Gaseous Formaldehyde Detection Based on a Closed-Microfluidic-Circuit Analysis,” *Chemosensors*, vol. 8, no. 3, p. 57, 2020.

[17] D. Mariuta *et al.*, “Optofluidic formaldehyde sensing: Towards on-chip integration,” *Micromachines (Basel)*, vol. 11, no. 7, p. 673, 2020.

[18] L. Mugherli, A. Lety-Stefanska, N. Landreau, R. F.-X. Tomasi, and C. N. Baroud, “Quantifying the sol–gel process and detecting toxic gas in an array of anchored microfluidic droplets,” *Lab Chip*, vol. 20, no. 2, pp. 236–243, 2020.

[19] X.-L. Guo, Y. Chen, H.-L. Jiang, X.-B. Qiu, and D.-L. Yu, “Smartphone-based microfluidic colorimetric sensor for gaseous formaldehyde determination with high sensitivity and selectivity,” *Sensors*, vol. 18, no. 9, p. 3141, 2018.

[20] P. Su *et al.*, “Monolithic on-chip mid-IR methane gas sensor with waveguide-integrated detector,” *Appl Phys Lett*, vol. 114, no. 5, p. 51103, 2019.

[21] B. D. Piorek, S. J. Lee, J. G. Santiago, M. Moskovits, S. Banerjee, and C. D. Meinhart, “Free-surface microfluidic control of surface-enhanced Raman spectroscopy for the optimized detection of airborne molecules,” *Proceedings of the National Academy of Sciences*, vol. 104, no. 48, pp. 18898–18901, 2007.

[22] K. Yang *et al.*, “Array-Assisted SERS Microfluidic Chips for Highly Sensitive and Multiplex Gas Sensing,” *ACS Appl Mater Interfaces*, vol. 12, no. 1, pp. 1395–1403, Jan. 2020, doi: 10.1021/acsami.9b19358.

[23] K. Yang *et al.*, “Ti3C2T x MXene-Loaded 3D Substrate toward On-Chip Multi-Gas Sensing with Surface-Enhanced Raman Spectroscopy (SERS) Barcode Readout,” *ACS Nano*, vol. 15, no. 8, pp. 12996–13006, 2021.

[24] A. Meckes, J. Behrens, O. Kayser, W. Benecke, Th. Becker, and G. Müller, “Microfluidic system for the integration and cyclic operation of gas sensors,” *Sens Actuators A Phys*, vol. 76, no. 1, pp. 478–483, 1999, doi: https://doi.org/10.1016/S0924-4247(99)00060-6.

[25] T. Becker, S. Mühlberger, C. Bosch-v.Braunmühl, G. Müller, A. Meckes, and W. Benecke, “Microreactors and microfluidic systems: an innovative approach to gas sensing using tin oxide-based gas sensors,” *Sens Actuators B Chem*, vol. 77, no. 1, pp. 48–54, 2001, doi: https://doi.org/10.1016/S0925-4005(01)00671-2.

[26] Y. Ueno, T. Horiuchi, O. Niwa, H. Zhou, T. Yamada, and I. Honma, “Improvement of on-site microfluidic benzene, toluene, xylene (BTX) gas sensor loaded with nanostructured mesoporous silicate,” *Sensors and materials*, vol. 15, no. 8, pp. 393–402, 2003.

[27] Y. Ueno, A. Tate, O. Niwa, H.-S. Zhou, T. Yamada, and I. Honma, “High benzene selectivity of mesoporous silicate for BTX gas sensing microfluidic devices,” *Anal Bioanal Chem*, vol. 382, no. 3, pp. 804–809, 2005.

[28] S. Chatterjee *et al.*, “Hydrogen gas sensing using aluminum doped ZnO metasurfaces,” *Nanoscale Adv*, vol. 2, no. 8, pp. 3452–3459, Aug. 2020, doi: 10.1039/D0NA00289E.

[29] F. Hossein-Babaei and V. Ghafarinia, “Gas analysis by monitoring molecular diffusion in a microfluidic channel,” *Anal Chem*, vol. 82, no. 19, pp. 8349–8355, 2010.

[30] V. Martini, S. Bernardini, M. Bendahan, K. Aguir, P. Perrier, and I. Graur, “Fabrication and characterization of gas detection microfluidic system,” *Procedia Eng*, vol. 5, pp. 1188–1191, 2010, doi: https://doi.org/10.1016/j.proeng.2010.09.324.

[31] V. Martini, S. Bernardini, M. Bendahan, K. Aguir, P. Perrier, and I. Graur, “Microfluidic gas sensor with integrated pumping system,” *Sens Actuators B Chem*, vol. 170, pp. 45–50, 2012, doi: <https://doi.org/10.1016/j.snb.2011.01.011>.

[32] V. Ghafarinia and F. Hossein-Babaei, “Single Sensor Gas Analysis Using a Microfluidic Channel,” *Key Eng Mater*, vol. 495, pp. 302–305, 2011.

[33] S. H. Lee, J. H. Lim, J. Park, S. Hong, and T. H. Park, “Bioelectronic nose combined with a microfluidic system for the detection of gaseous trimethylamine,” *Biosens Bioelectron*, vol. 71, pp. 179–185, 2015, doi: <https://doi.org/10.1016/j.bios.2015.04.033>.

[34] F. Hossein-Babaei, A. Hooshyar Zare, V. Ghafarinia, and S. Erfantalab, “Identifying volatile organic compounds by determining their diffusion and surface adsorption parameters in microfluidic channels,” *Sens Actuators B Chem*, vol. 220, pp. 607–613, 2015, doi: <https://doi.org/10.1016/j.snb.2015.06.014>.

[35] M. M. Montazeri *et al.*, “A sensor for nuisance sewer gas monitoring,” in *2017 IEEE SENSORS*, 2017, pp. 1–3. doi: 10.1109/ICSENS.2017.8234327.

[36] M. Paknahad, C. Mcintosh, and M. Hoorfar, “Selective detection of volatile organic compounds in microfluidic gas detectors based on ‘like dissolves like,’” *Sci Rep*, vol. 9, no. 1, p. 161, 2019, doi: 10.1038/s41598-018-36615-6.

[37] S. Janfaza *et al.*, “A Nanostructured Microfluidic Artificial Olfaction for Organic Vapors Recognition,” *Sci Rep*, vol. 9, no. 1, p. 19051, 2019, doi: 10.1038/s41598-019-55672-z.

[38] M. Ghazi, S. Janfaza, H. Tahmooressi, N. Tasnim, and M. Hoorfar, “Selective detection of VOCs using microfluidic gas sensor with embedded cylindrical microfeatures coated with graphene oxide,” *J Hazard Mater*, vol. 424, p. 127566, 2022, doi: https://doi.org/10.1016/j.jhazmat.2021.127566.

[39] M. Ghazi *et al.*, “Enhanced selectivity of microfluidic gas sensors by modifying microchannel geometry and surface chemistry with graphene quantum dots,” *Sens Actuators B Chem*, vol. 342, p. 130050, 2021, doi: https://doi.org/10.1016/j.snb.2021.130050.

[40] M. Ghazi, N. Tasnim, and M. Hoorfar, “Selective monitoring of natural gas sulphur-based odorant mixture of t-butyl mercaptan and methyl ethyl sulphide using an array of microfluidic gas sensors,” *J Hazard Mater*, vol. 438, p. 129548, 2022, doi: https://doi.org/10.1016/j.jhazmat.2022.129548.

[41] W. Raj, D. Yang, and C. Priest, “Rapid Fabrication of Superhydrophobic Virtual Walls for Microfluidic Gas Extraction and Sensing,” *Micromachines (Basel)*, vol. 12, no. 5, p. 514, 2021.

[42] J. W. Grate, B. Liu, R. T. Kelly, N. C. Anheier, and T. M. Schmidt, “Microfluidic sensors with impregnated fluorophores for simultaneous imaging of spatial structure and chemical oxygen gradients,” *ACS Sens*, vol. 4, no. 2, pp. 317–325, 2019.

[43] F. Bunge *et al.*, “Microfluidic oxygen sensor system as a tool to monitor the metabolism of mammalian cells,” *Sens Actuators B Chem*, vol. 289, pp. 24–31, 2019.

[44] M. Paknahad, J. S. Bachhal, A. Ahmadi, and M. Hoorfar, “Characterization of channel coating and dimensions of microfluidic-based gas detectors,” *Sens Actuators B Chem*, vol. 241, pp. 55–64, 2017.

[45] S. M. Grist, J. C. Schmok, M.-C. A. Liu, L. Chrostowski, and K. C. Cheung, “Designing a microfluidic device with integrated ratiometric oxygen sensors for the long-term control and monitoring of chronic and cyclic hypoxia,” *Sensors*, vol. 15, no. 8, pp. 20030–20052, 2015.

[46] M. Paknahad, J. S. Bachhal, A. Ahmadi, and M. Hoorfar, “Highly selective multi-target 3D-printed microfluidic-based breath analyzer,” in *2016 IEEE 29th International Conference on Micro Electro Mechanical Systems (MEMS)*, 2016, pp. 905–908.

[47] M. Paknahad, V. Ghafarinia, and F. Hossein-Babaei, “A microfluidic gas analyzer for selective detection of biomarker gases,” in *2012 IEEE Sensors Applications Symposium Proceedings*, 2012, pp. 1–5.

[48] M. Aghaseyedi, A. Salehi, S. Valijam, and M. Shooshtari, “Gas Selectivity Enhancement Using Serpentine Microchannel Shaped with Optimum Dimensions in Microfluidic-Based Gas Sensor,” *Micromachines 2022, Vol. 13, Page 1504*, vol. 13, no. 9, p. 1504, Sep. 2022, doi: 10.3390/MI13091504.

[49] V. Nock, R. J. Blaikie, and T. David, “Patterning, integration and characterisation of polymer optical oxygen sensors for microfluidic devices,” *Lab Chip*, vol. 8, no. 8, pp. 1300–1307, 2008.

[50] B. H. Timmer, M. van Delft, W. W. Koelmans, W. Olthuis, and A. van den Berg, “Selective low concentration ammonia sensing in a microfluidic lab-on-a-chip,” *IEEE Sens J*, vol. 6, no. 3, pp. 829–835, 2006.
